# Climate change influences on the potential geographic distribution of the disease vector tick *Ixodes ricinus*

**DOI:** 10.1371/journal.pone.0189092

**Published:** 2017-12-05

**Authors:** Abdelghafar A. Alkishe, A. Townsend Peterson, Abdallah M. Samy

**Affiliations:** 1 Biodiversity Institute, University of Kansas, Lawrence, Kansas, United States of America; 2 Zoology Department, Faculty of Science, University of Tripoli, Tripoli, Libya; 3 Entomology Department, Faculty of Science, Ain Shams University, Abbassia, Cairo, Egypt; University of Toledo College of Medicine and Life Sciences, UNITED STATES

## Abstract

**Background:**

*Ixodes ricinus* is a species of hard tick that transmits several important diseases in Europe and North Africa, including Lyme borreliosis and tick-borne encephalitis. Climate change is affecting the geographic distributions and abundances of arthropod vectors, which in turn influence the geographic distribution and epidemiology of associated vector-borne diseases. To date, few studies have investigated effects of climate change on the spatial distribution of *I*. *ricinus* at continental extents. Here, we assessed the potential distribution of *I*. *ricinus* under current and future climate conditions to understand how climate change will influence the geographic distribution of this important tick vector in coming decades.

**Method:**

We used ecological niche modeling to estimate the geographic distribution of *I*. *ricinus* with respect to current climate, and then assessed its future potential distribution under different climate change scenarios. This approach integrates occurrence records of *I*. *ricinus* with six relevant environmental variables over a continental extent that includes Europe, North Africa, and the Middle East. Future projections were based on climate data from 17 general circulation models (GCMs) under 2 representative concentration pathway emissions scenarios (RCPs), for the years 2050 and 2070.

**Result:**

The present and future potential distributions of *I*. *ricinus* showed broad overlap across most of western and central Europe, and in more narrow zones in eastern and northern Europe, and North Africa. Potential expansions were observed in northern and eastern Europe. These results indicate that *I*. *ricinus* populations could emerge in areas in which they are currently lacking, posing increased risks to human health in those areas. However, the future of *I*. *ricinus* ticks in some important regions such the Mediterranean was unclear owing to high uncertainty in model predictions.

## Introduction

*Ixodes ricinus* is the most common arthropod vector of human disease in Europe and nearby regions [1]. This tick species infests a wide variety of wild vertebrate species, as well as other accidental hosts, such as humans, livestock, and companion animals [2]. *Ixodes ricinus* transmits a wide variety of tick-borne pathogens, including the spirochete bacteria of the *Borrelia burgdorferi* sensu lato (sl) species complex and the tick-borne encephalitis virus. These pathogens are transmitted to humans mostly by bites of immature nymphs, which are more abundant, smaller and therefore harder to notice than adult ticks [3]. Some species of the *B*. *burgdorferi* sl complex cause Lyme disease (or Lyme borreliosis, LB) and tick-borne encephalitis virus causes tick-borne encephalitis (TBE). LB is prevalent in Central Europe where the highest infection rates have been recovered from ixodid ticks [4]. TBE occurs in eastern, western, and central Europe, as well as in Russia [5, 6]. Active surveillance of both diseases in Europe indicates a need to study the distributional ecology of their vector, *I*. *ricinus*. It also emphasizes the importance of understanding likely effects of global warming on the distribution of this vector species.

The geographic distribution of *I*. *ricinus* is related to climate factors such as humidity, soil water, and air temperature, and to vegetation type, land use, and disturbance [7]. Some studies have found that the latitudinal and elevational limits of *I*. *ricinus* have shifted with increasing global temperatures [8–10]. Over the last century, annual mean temperature has risen 0.7°C globally, and another 1.1°C increase is expected in the 21^st^ century [11]. This warming is expected to influence vectors and reservoir hosts, in turn affecting the epidemiology of the vector-borne pathogens [11, 12]. Global warming is expected to alter vector development, vector physiology and fitness, geographic distribution of vectors and hosts, and vector-host-pathogen interactions [12]. Several recent publications have presented predictions of how climate change will alter the distribution of vector-borne diseases transmitted by ticks, sandflies, and mosquitoes. These studies investigate how changes in spatial distributions of arthropod vectors may influence future spatial distributions of vector-borne pathogens [13, 14].

Modeling the ecological requirements of species to anticipate future disease transmission patterns is challenging [11]. Previous studies of the potential distribution of *I*. *ricinus* have generally covered small geographic areas [15, 16], such as some studies in single countries that have attempted to understand the population dynamics of this species [17, 18]. A recent paper [19] studied effects of global climate change on *I*. *ricinus* across its range, but used older climate scenarios (4^th^ generation Intergovernmental Panel on Climate Change (IPCC), emissions scenarios A2 and B2) from one general circulation model (GCM) only for projection. Here, we prepared a data set of *I*. *ricinus* occurrences that covered its entire geographic range in Europe and North Africa, and we carefully removed bias that might affect model predictions. We used a maximum entropy algorithm to estimate the ecological niche of *I*. *ricinus*, and transferred this model onto future conditions for the years 2050 and 2070 under 17 GCMs for two representative concentration pathway (RCP) scenarios for greenhouse gases. We thus present the most comprehensive models developed to date for this important disease vector, and explore their implications under the newest suite of future climate scenarios.

## Materials and methods

### Input data

Primary occurrence records for *I*. *ricinus* were obtained from diverse sources. Data were drawn from the Global Biodiversity Information Facility (GBIF; www.gbif.org; ~2110 occurrence points), VectorMap (www.vectormap.org; ~1801 occurrence points), and the scientific literature [20] (~1195 points; S1 File). Sampling was concentrated in Great Britain and Germany thanks to surveillance by the European Vector Map Program of the European Center for Disease Prevention and Control (ECDC; http://ecdc.europa.eu/en/healthtopics/vector/vector-maps/). The initial set of occurrence records was subjected to several data cleaning steps to reduce possible biases in calibrating ecological niche models (ENMs) [21]. (1) We discarded all records with unknown geographic references, and removed all duplicate records. (2) The data were further filtered by distance, so that all redundant records occurring in a single 10’ cell (~20 km) were omitted. (3) Finally, we accounted for marked differences in sampling density across countries: data records were filtered by balancing the density of occurrences on a country-by-country basis. We chose Spain as a reasonable intermediate-density reference point (6 occurrence records /100,000 km^2^) to overcome problems associated with oversampling or undersampling observed in some countries. Although we discarded large numbers of data points, this step removes large-scale spatial biases, and allows a better estimation of niche characteristics [22].

**Fig 1:**
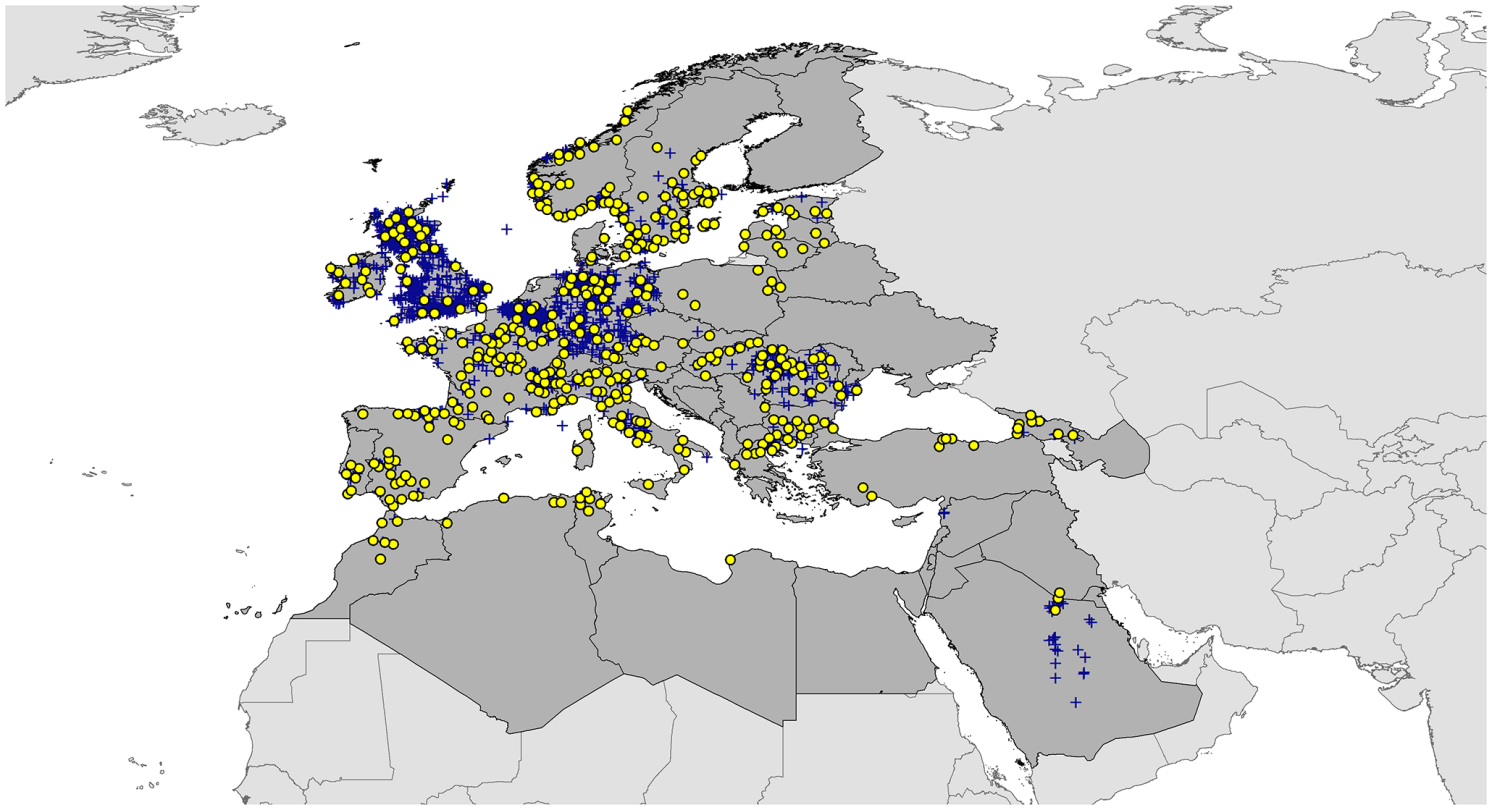
Occurrence records of *Ixodes ricinus* derived from various sources. Blue crosses indicate the original set of occurrence records; yellow circles are occurrence records retained after filtering the data.

The final balanced dataset of *I*. *ricinus* included 416 occurrence points, which we separated five times randomly into equal-sized subsets of 208 points, one subset was for model calibration and the other for model evaluation (Fig 1). These 5 random subgroups provide replicate views of model results and give a better idea of the variation resulting from the availability of occurrence data.

We obtained data on 19 “bioclimatic” variables from the WorldClim climate data version 1.4 [23] available via www.worldclim.org. These variables were derived from interpolation of average monthly temperature and rainfall data obtained from weather stations during 1950–2000. We removed variables 8–9 and 18–19 because of known spatial artefacts. We used the data layers at 10’ spatial resolution because of the continental extent of our models. We obtained parallel data layers for 17 general circulation models (GCMs; Table 1) for each representative concentration pathway (RCP) for each time period. We chose two representative concentration pathways, RCP 4.5 and RCP 8.5 (corresponding to lower and higher greenhouse gas emissions, respectively) for 2050 and 2070 to account for possible climate change influences in both scenarios and in two different times. We used diverse GCMs available from the WorldClim archive to estimate both the future distributional potential of *I*. *ricinus* based on each individual GCM, which was a key element in assessing uncertainty in predictions deriving from GCM choice.

**Table 1:** Summary of general circulation models (GCMs) explored in our analysis.

GCM	Code	Modeling center or group
ACCESS 1–0	AC	Commonwealth Scientific and Industrial Research Organization (CSIRO) and Bureau of Meteorology (BOM), Australia
BCC-CSM 1–1	BC	Beijing Climate Center, China Meteorological Administration
CCSM4	CC	National Center for Atmospheric Research, USA
CNRM-CM 5	CN	Centre National de Recherches Météorologiques, France
GFDL-CM 3	GF	NOAA Geophysical Fluid Dynamics Laboratory, USA
GISS-E2-R	GS	NASA Goddard Institute for Space Studies, USA
HadGEM 2-AO	HD	National Institute of Meteorological Research, Korea Meteorological Administration
HadGEM 2-ES	HE	Met Office Hadley Centre (additional realizations from Instituto Nacional de Pesquisas Espaciais)
HadGEM 2-CC	HG	Met Office Hadley Centre (additional realizations from Instituto Nacional de Pesquisas Espaciais).
INMCM4	IN	Institute for Numerical Mathematics, Russia
IPSL-CM5A-LR	IP	Institute Pierre-Simon Laplace, France
MIROC-ESM-CHEM	MI	Japan Agency for Marine-Earth Science and Technology, Atmosphere and Ocean Research Institute, and National Institute for Environmental Studies, Japan
MIROC-ESM	MR	Japan Agency for Marine-Earth Science and Technology, Atmosphere and Ocean Research Institute, and National Institute for Environmental Studies, Japan
MIROC5	MC	Atmosphere and Ocean Research Institute, National Institute for Environmental Studies, and Japan Agency for Marine-Earth Science and Technology, Japan
MPI-ESM-LR	MP	Max-Planck-Institut für Meteorolgie, Germany
MRI-CGCM3	MG	Meteorological Research Institute, Japan
NorESM 1-M	NO	Norwegian Climate Centre, Norway

## Ecological niche modeling

Ecological niche models (ENMs) were estimated based on the maximum entropy algorithm implemented in Maxent 3.3.3k [24]. We first used the jackknifing function in Maxent to identify the most important set of environmental variables. We used SDMTools in ArcGIS 10.3 to remove variables with high inter-variable correlations. In the end, we used 6 variables for analysis: (1) annual mean temperature, (2) mean diurnal temperature range, (3) isothermality, (4) annual temperature range, (5) annual precipitation, and (6) precipitation seasonality. Associations between presence points and environmental variables [25] were used to reconstruct the ecological niche of *I*. *ricinus*. We hypothesized an accessible area (**M** in the BAM diagram framework) [26] that included all of Europe, North Africa, and parts of the Middle East but excluded western Asia for lack of data documenting *I*. *ricinus* occurrence in this region. We used Maxent’s bootstrap function to create 10 replicate analyses. We used a partial receiver operating characteristic (pROC) [27] to test model robustness via Niche Toolbox (http://shiny.conabio.gob.mx:3838/nichetoolb2/); the 5 testing subsets of the occurrence data were used to test model predictions. For further evaluation of our predictions regarding the occurrence of *I*. *ricinus*, we used a set of 3186 *I*. *ricinus* records discarded during the early phases of data filtering of the original dataset. We excluded those records used in model calibration; we used a one-tailed cumulative binomial probability test to assess the probability of obtaining the observed level of correct predictions by chance alone given the background expectation of correct predictions determined by the proportional coverage of the study area by regions of predicted suitability.

To summarize the model results for present-day conditions, we calculated the median of the medians from the predictions based on the 5 subsets of occurrences as an estimate of the current geographic distribution of *I*. *ricinus*. For future conditions, we calculated medians across all single-GCM median model outputs (5 subsets of occurrences x 17 GCMs = 85 combinations), as an estimate of the potential distribution of *I*. *ricinus* under each corresponding RCP. For present-day conditions, an uncertainty index was derived from the range (maximum—minimum) of predictions from 5 Maxent runs (i.e. 5 combinations based on the 5 subsets of occurrence records), which summarizes uncertainty deriving from the particular availability of occurrence data. For the future conditions, an uncertainty index of future model predictions was calculated as the range across all combinations of GCMs and occurrence subsets (within each RCP; 5 subsets of occurrences x 17 GCMs = 85 combinations) [14], thereby including uncertainty deriving both from the availability of occurrence data and variation among GCMs in future climates anticipated. To avoid known problems with model overfitting [28], we thresholded models using a fixed allowable omission error rate of *E* = 5% [29], assuming that 5% of the occurrence data may have included errors that misrepresented environmental values. This thresholding approach omitted the 5% of records with the lowest suitability to account for the reality that such large datasets often include some errors (either in the occurrence data or the environmental data), so this 5% trimming allows those errors to be ignored and not affect the results.

Mobility-oriented parity (MOP) was used to assess the degree of novelty of climate conditions under all future climate scenarios (i.e., 17 GCMs x 2 RCPs x 2 time periods) relative to present-day conditions. This novelty is actually a distance in environmental space between the environmental characteristics of the site in question and the set of environments represented across the reference region. In the present study, the site in question is generally in a future time period, and the reference region is in the present. MOP evaluates the general novelty of conditions, and highlights regions where strict extrapolation (i.e. values outside of the range of environments in the reference region/time) occurs, to give a view of certainty and uncertainty across various sectors of the region of interest [30]. Any extrapolative transfer of the model should be interpreted with considerable caution. To summarize MOP results within each RCP, we counted the number of GCMs for which each pixel was strictly extrapolative, and also calculated the average distance from the reference (present) conditions.

## Results

After detailed data cleaning and spatial balancing, our initial total of 5107 occurrence points for *I*. *ricinus* from diverse sources was filtered and reduced to 416 spatially unique points at 10’ resolution. This data filtering reduced problems associated with artificial clumping of occurrence records, which is related to biases in sampling and reporting (Fig 1). Calibrating models for *I*. *ricinus* based on the 5 subgroups of occurrence points yielded predictions that gave area under the curve (AUC) ratios above null expectations in all five partial ROC analyses (*P* < 0.001; S2 File). The *I*. *ricinus* model successfully anticipated 3110 records out of the additional 3186 test records, which was significantly better than random expectations (P < 0.001; S3 File). Annual precipitation, annual temperature range, and annual mean temperature were the most influential factors and contributed >86% to the Maxent model (S4 File).

**Fig 2:**
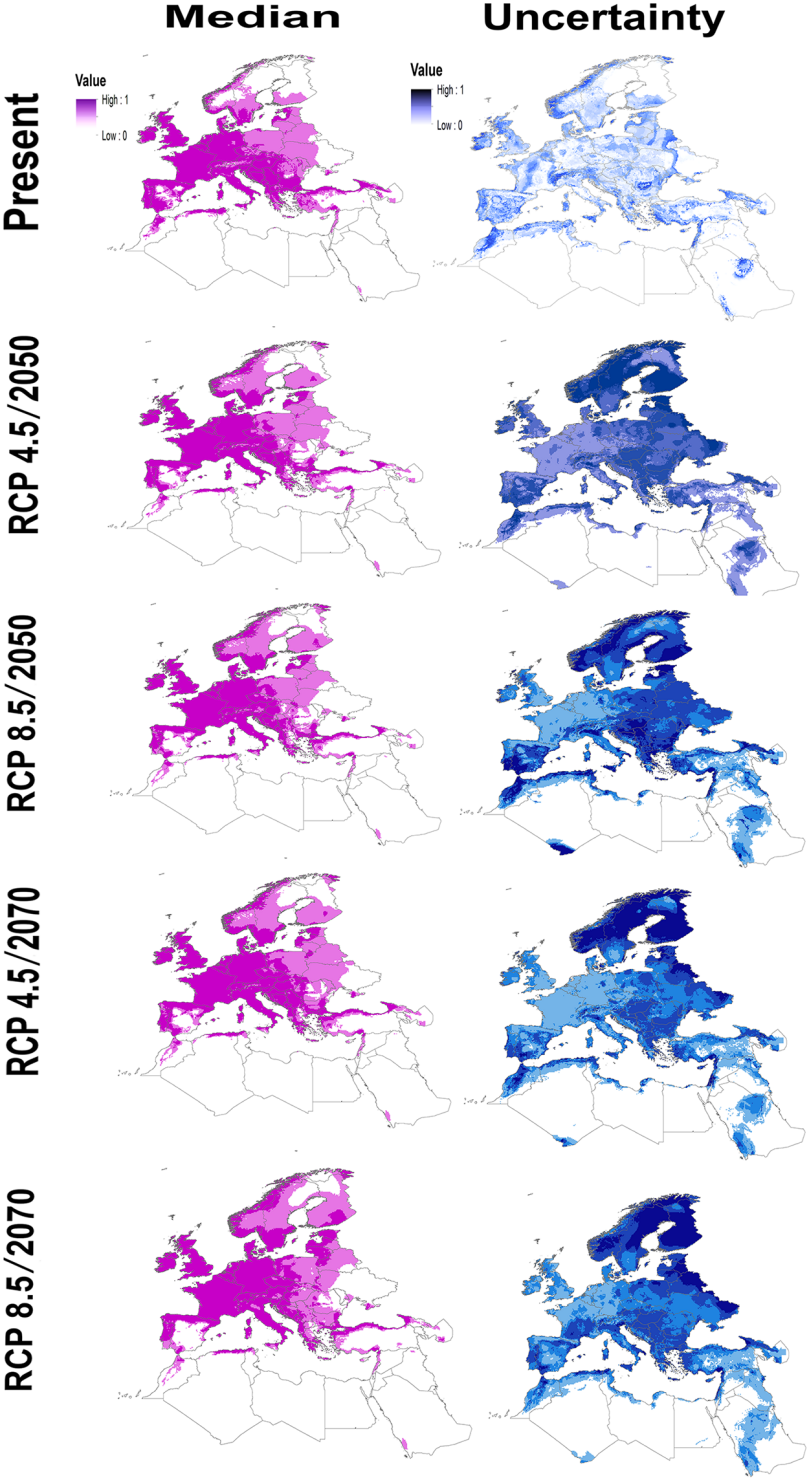
Current and future potential distribution of *Ixodes ricinus* based on present-day and future climatic conditions. Left-hand maps show potential distributions whereas right-hand maps indicate the uncertainty.

Models based on present-day conditions revealed areas with high suitability for *I*. *ricinus* across Central and Western Europe including the countries of Great Britain, France, Germany, Belgium, the Netherlands, Greece, and Italy. In Northern Europe, the highest suitability for *I*. *ricinus* was in southern Finland, southern Sweden, and western Norway. Suitable areas also occurred in western Turkey, the Middle East, and in more restricted areas in Morocco, Algeria, and Tunisia. Transferring models to future conditions, the present-day and future distributional patterns largely coincided. However, our model predictions indicated some potential for expansion into areas not identified as suitable for *I*. *ricinus* under present-day conditions, particularly in Northern Europe (Fig 2), albeit with high uncertainty with respect to the potential distributions in both present and future.

Under present-day conditions, low uncertainty (deriving from availability of occurrence data) was observed across the study area (which can be uncertainty as regards prediction of presence or prediction of absence), except some areas in Morocco, Ireland, and eastern Norway (Fig 2). In the future, uncertainty with respect to predictions of geographic distribution of *I*. *ricinus* and deriving from both availability of occurrence data and variation among GCMs in future conditions anticipated was observed in Scandinavia and parts of Eastern Europe. Under future conditions, the levels of uncertainty varied among RCP scenarios and time periods, but this variation was not necessarily related to the density of the available occurrence data. For RCP 4.5 in 2050, high uncertainty was concentrated in southern Finland, central Norway, and Sweden, and medium uncertainty was present in parts of Eastern Europe and North Africa, whereas lower uncertainty was in Western Europe and the Middle East. For RCP 8.5 for the 2050s, high uncertainty was restricted to southern Finland, eastern Sweden, southern Spain, and northern Morocco; low uncertainty areas were in Western Europe and the Middle East. For RCP 4.5 for the 2070s, models showed high uncertainty in Finland, Norway, central and northern Sweden, and eastern Belarus. RCP 8.5 for the 2070s showed high uncertainty in Finland, eastern Sweden, eastern Belarus, and eastern Ukraine; low uncertainty was in Western Europe, North Africa, and the Middle East (Fig 2).

**Fig 3:**
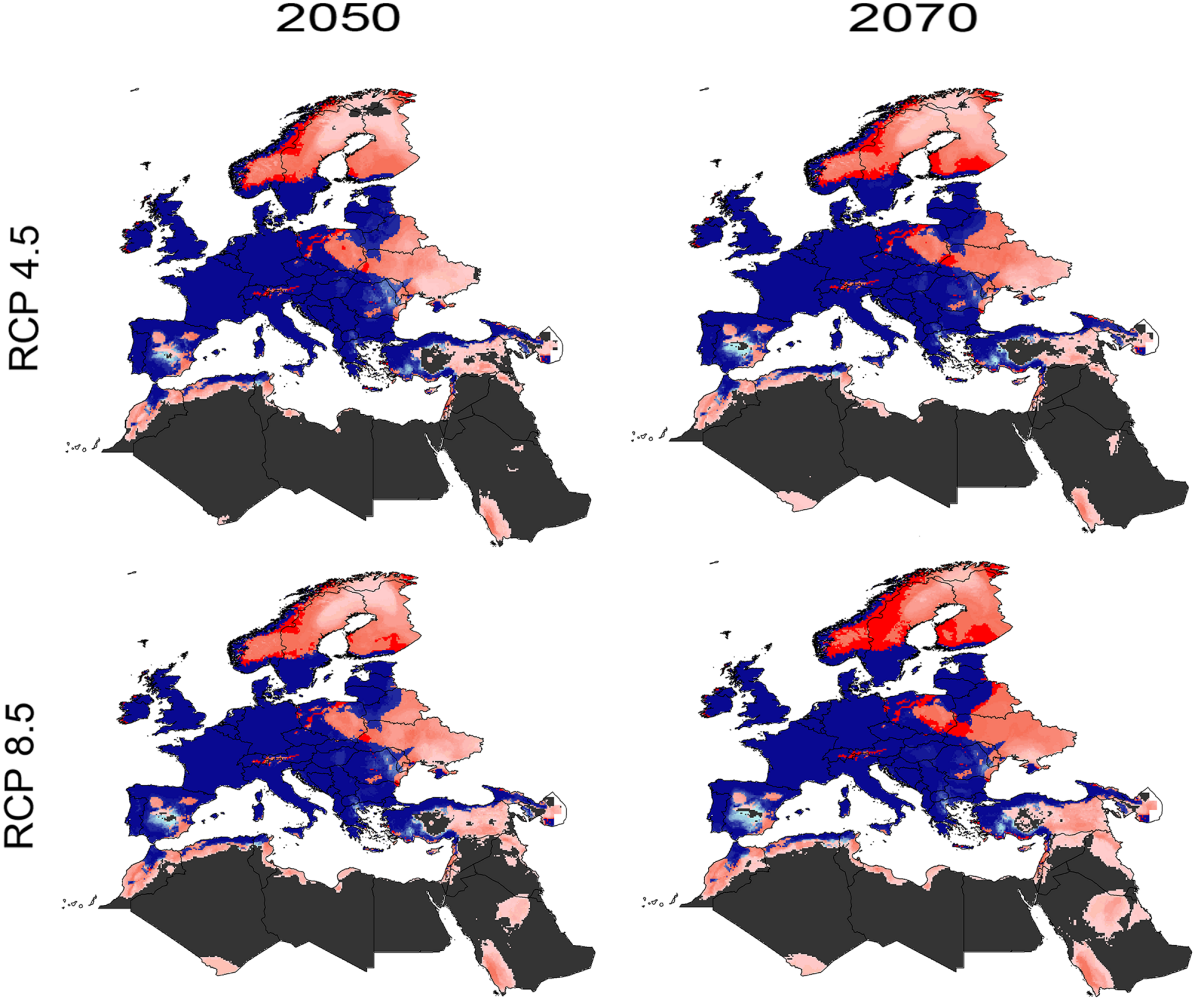
Summary of the binary modeled potential distributions of *Ixodes ricinus* under future conditions to show suitable areas and to illustrate differences between representative concentration pathways (RCPs) and time periods. Blue color indicates model suitability under both present and future suitability (light blue denotes low confidence and dark blue denotes high confidence), red color represents predicted expansion areas in the future suitability (light red = low confidence, dark red = high confidence); dark gray areas are not suitable.

Binary (thresholded) predictions for future conditions showed differences between RCP 4.5 and RCP 8.5 in 2050 and 2070 (Fig 3). In terms of agreement between present and future conditions, range expansion was indicated in North Africa in the coastal regions of Morocco, Algeria, and Tunisia. Under RCP 4.5 for the 2050s, expansions are expected (although with low confidence) in North Africa, the Middle East, and Eastern and Northern Europe. Under RCP 8.5 for the 2050, the expansions in Eastern and Northern Europe, Turkey, and the Middle East were predicted to be wider compared to RCP 4.5 for 2050. In 2070, suitable areas for *I*. *ricinus* increased in Norway, Sweden, and Finland, with high confidence in Turkey, the Middle East and North Africa under RCP 4.5 and RCP 8.5 (Fig 3). Between the present-day and 2050, the potential distribution area of *I*. *ricinus* increased by 10.8% and 11.7% under RCP 4.5, and RCP 8.5, respectively. Between the present day and 2070, the potential distributional area of *I*. *ricinus* is anticipated to increase by 11.5% and 14.5% under RCP 4.5, and RCP 8.5, respectively. Thus, global warming is expected to increase the geographic distribution of *I*. *ricinus* in Europe, North Africa, and the Middle East. Compared to RCP 4.5, the potential distributional area under RCP 8.5 increased by 0.9% and 3.0% for 2050 and 2070, respectively; a summary matrix (S5 File) and detailed maps of potential distributions under each individual GCM are provided in the supplementary materials (S6 File); the final future projections are summarized as a GeoTIFF data file at https://doi.org/10.6084/m9.figshare.5067373.

**Fig 4:**
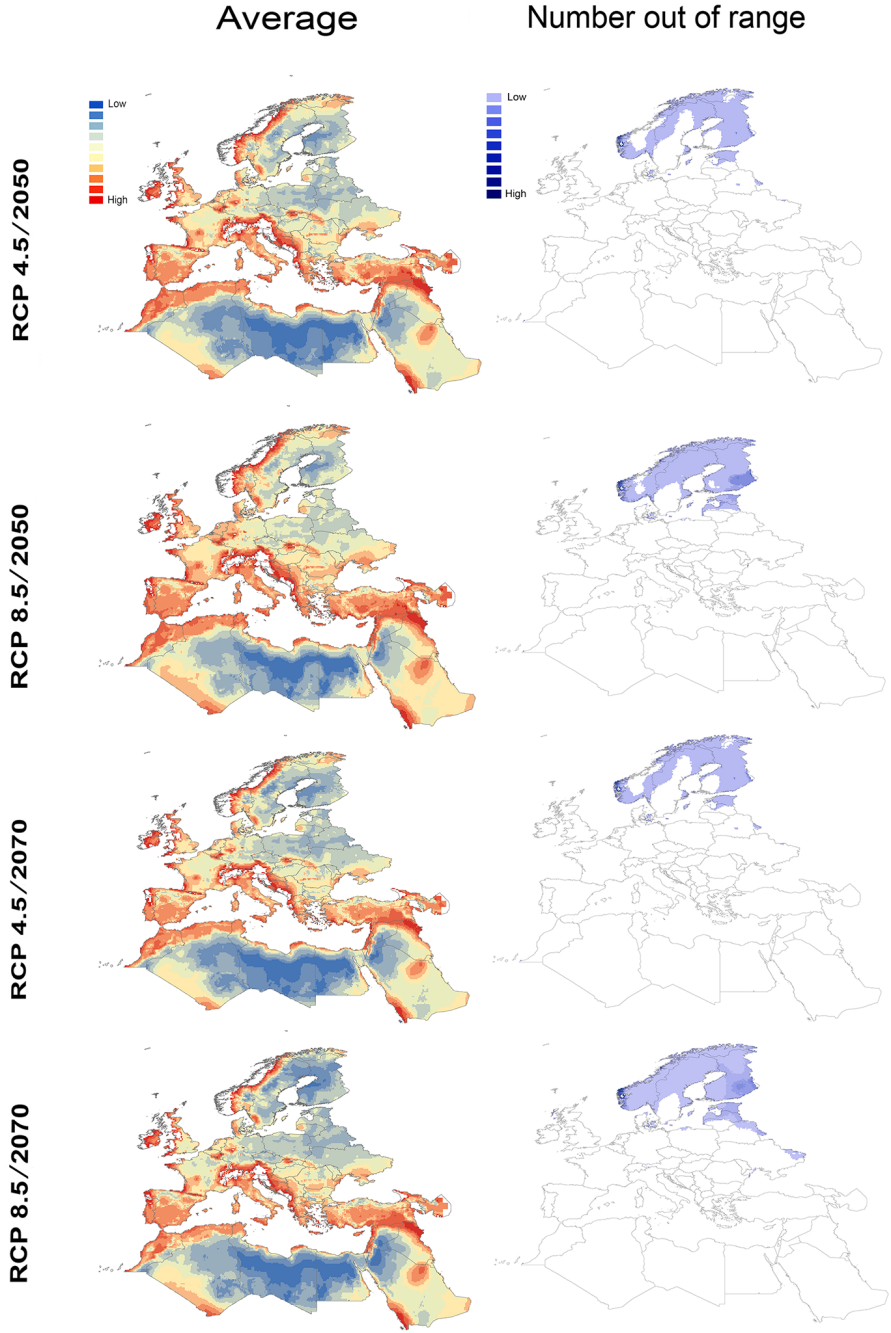
MOP calculations for model transfers from present to future climate scenarios for 17 GCMs (RCP 4.5 and RCP 8.5) in 2050 and 2070. Left-hand panels show the average MOP distance among models (dark red represents high average and dark blue represents low average). Right-hand panels show the number of models out of range (dark blue represents areas with most frequent strict extrapolation).

MOP results indicated high novelty of future conditions along the entire Mediterranean rim of southern Europe and North Africa, as well as in northern Scandinavia (Fig 4). Under RCP 4.5, MOP detected extrapolative conditions in as many as 10 of 17 models in both 2050 and 2070. Under RCP 8.5, MOP detected extrapolative conditions in 11 and 13 of 17 models in 2050 and 2070, respectively. Strict extrapolation was manifested chiefly in northern extremes of Scandinavian countries.

## Discussion

Tick-borne diseases such as TBE and LB are greatly influenced by tick ecology and other factors such as habitat structure, climate, human activities, and the community of vertebrate species that are reservoir hosts for the tick-borne pathogens [31]. Several studies have suggested that increasing temperatures will affect the geographic distribution and ecology of *I*. *ricinus* in Europe [19, 32, 33]. Climate change may affect the seasonal activities and feeding behavior of the different life stages of *I*. *ricinus* [34, 35]. For instance, rising temperatures could lead to milder winter conditions, extending spring and fall seasons in northern regions, making them more suitable for *I*. *ricinus*. Indeed, expansions of northern distributional limits have been reported for this species in Norway and Sweden since the 1980s [8, 36, 37]. Elevational range expansions of *I*. *ricinus* have been recorded in the Czech Republic and Switzerland [38]. Our results also showed that the presence of *I*. *ricinus* will increase in the European Alps with high certainty. Increased TBE transmission in Europe during the last 2 decades has been attributed to climate change, socio-economic changes, and anthropogenic activities [39].

Several factors must be considered before interpreting our model predictions regarding the expansions and changes in the potential distribution of *I*. *ricinus*. First, as with all ixodid ticks, *I*. *ricinus* spends most of its life cycle off the host and in the environment, so climate change may have direct effects on its abundance and distribution [40]. Second, other abiotic factors (e.g. land use, soil characteristics), and biotic factors (e.g., host abundance and competition with other species), should be considered in tandem with climate effects [40–44]. Third, newly suitable areas must be accessible to *I*. *ricinus* via dispersal for actual range expansions to take place [45]. For example, migratory birds are potential agents of tick dispersal across new regions [46]. Migratory bird-mediated dispersal may allow *I*. *ricinus* to respond to improving conditions in some of the expansion areas, and create new biotic combinations suitable for circulation of tick-borne pathogens.

Our results were similar to those of Porretta *et al*. [19] in terms of the distribution of *I*. *ricinus* presently extending across most of Europe and parts of North Africa and the Middle East. The two studies made similar future projections for *I*. *ricinus*: both anticipated potential for expansion into new areas in northern and eastern Europe. This study differs from Porretta *et al*. [19] in using 6 climate variables for analysis, the most updated RCPs, many more individual GCMs, and the IPCC 5^th^ Assessment scenarios for 2050 and 2070. Also, we used diverse data sources, and focused on Europe, North Africa, and the Middle East, to develop predictions of the potential distribution of this tick species into the future. We did not include western Asia in our study owing to a lack of sufficient occurrence data from this region. In addition, we included mobility-oriented parity (MOP) to understand certainty and uncertainty in different areas in the region of interest [30]. Thus, whereas the results of the two studies did not differ qualitatively, our work provides a clearer picture of certainty and uncertainty in these predictions for an important disease vector species.

Our models anticipated potential range expansions more broadly in northern Europe, with milder winter conditions as temperature increases [19]. In Sweden, for example, the climate has changed to be significantly warmer in the last 3 decades: the 8 warmest Novembers on record were between 2000 and 2009 [47]. These changes can allow more ticks to survive the winter, and increase the probability of tick bites [19]. Given that LB, TBE, and various other tick-borne diseases cause serious health problems, predicting future suitable areas for *I*. *ricinus* can help to guide plans to manage and mitigate effects of these public health threats [48].

## Supporting information

S1 File Primary occurrence records for *Ixodes ricinus* collected from diverse sources for the study. These records were drawn from the Global Biodiversity Information Facility (GBIF), VectorMap, and the scientific literature.
(CSV)

S2 File Detailed results of model evaluation for *Ixodes ricinus* ecological niche models. This supporting information describes the detailed results of model evaluation based on partial ROC tests applied to 5 random subsets of occurrence data.
(PDF)

S3 File Relationship of ecological niche model predictions to the distribution of 416 and 3186 records of *Ixodes ricinus* occurrences used for model calibration and testing, respectively. The testing records are those retained from the original occurrences (i.e. black dots) but none coincided with the 416 records used in model calibration (i.e. yellow circles).
(PDF)

S4 File Relative contributions of six environmental variables to the ecological niche models for *Ixodes ricinus* and its response to those variables. This supporting information shows the results regarding the relative contributions of six environmental variables to models for *I*. *ricinus* based on 5 random subsets of occurrences.
(PDF)

S5 File Summary matrix of potential distributions and range expansions of *Ixodes ricinus* based on binary (thresholded) versions of ecological niche model for each individual general circulation model (GCM) in this study. (CSV)

S6 File Predicted potential distribution maps for *Ixodes ricinus* under each individual general circulation model (GCM). (PDF)
