# Supplementary material for: Climate change influences on the potential geographic distribution of the disease vector tick Ixodes ricinus
Source: PLoS One. 2017 Dec 5;12(12):e0189092. doi: 10.1371/journal.pone.0189092 (PMC5716528; doi:10.1371/journal.pone.0189092)
Supplement: S2 File — This supporting information describes the detailed results of model evaluation based on partial ROC tests applied to 5 random subsets of occurrence data. (PDF) [file pone.0189092.s002.pdf]

S2 File. Detailed results of model evaluation for *Ixodes ricinus* ecological niche models. This supporting information describes the detailed results of model evaluation based on partial ROC tests applied to 5 random subsets of occurrence data.

Table I. Results of partial ROC analysis to test statistical significance of *Ixodes ricinus* predictions based on 5 random subsets of occurrences. Partial ROC (*p*ROC) tests evaluate the statistical significance of niche model predictions in comparison with a random classifier, subject to a maximum proportional amount of omission error: no. NS indicates the number out of 1000 random replicate analyses for which the AUC ratio was greater than 1, *p*ROC range and mean indicate the mean and range among all 1000 replicates, respectively.

Subset #	*p*ROC			
	no. NS	*p*ROC range	Mean	*p*-value
Subset #1	1000	1.602 – 1.728	1.664	< 0.0001*
Subset #2	1000	1.603 – 1.724	1.662	< 0.0001*
Subset #3	1000	1.619 – 1.729	1.672	< 0.0001*
Subset #4	1000	1.578 – 1.695	1.639	< 0.0001*
Subset #5	1000	1.591 – 1.711	1.659	< 0.0001*

*indicates significant models

Fig I: Predicted potential distribution of *Ixodes ricinus* with occurrence records used for model calibration (yellow circles) and occurrence records used for testing (black circles) using the partial receiver operating characteristics approach. The map shows the consistent agreement between the testing point and the prediction. We presented both sets of points to observe the difference between the two different sets of occurrences; the one used for model calibration and the one used for model testing. The maps are presented for the five random subsets; a) subset #1, b) subset #2, c) subset #3, d) Subset #4, and e) subset #5.

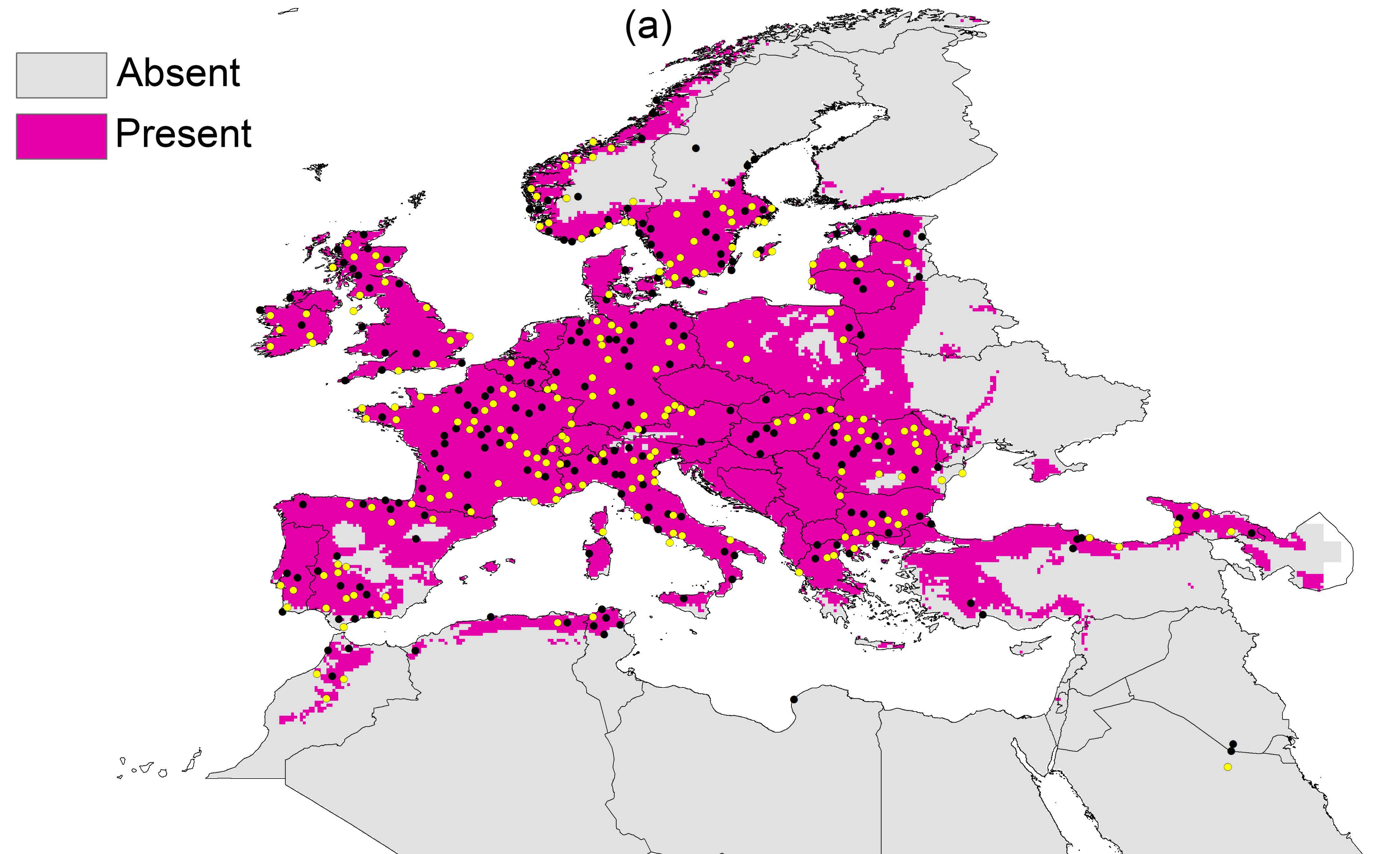
(a)
Absent
Present

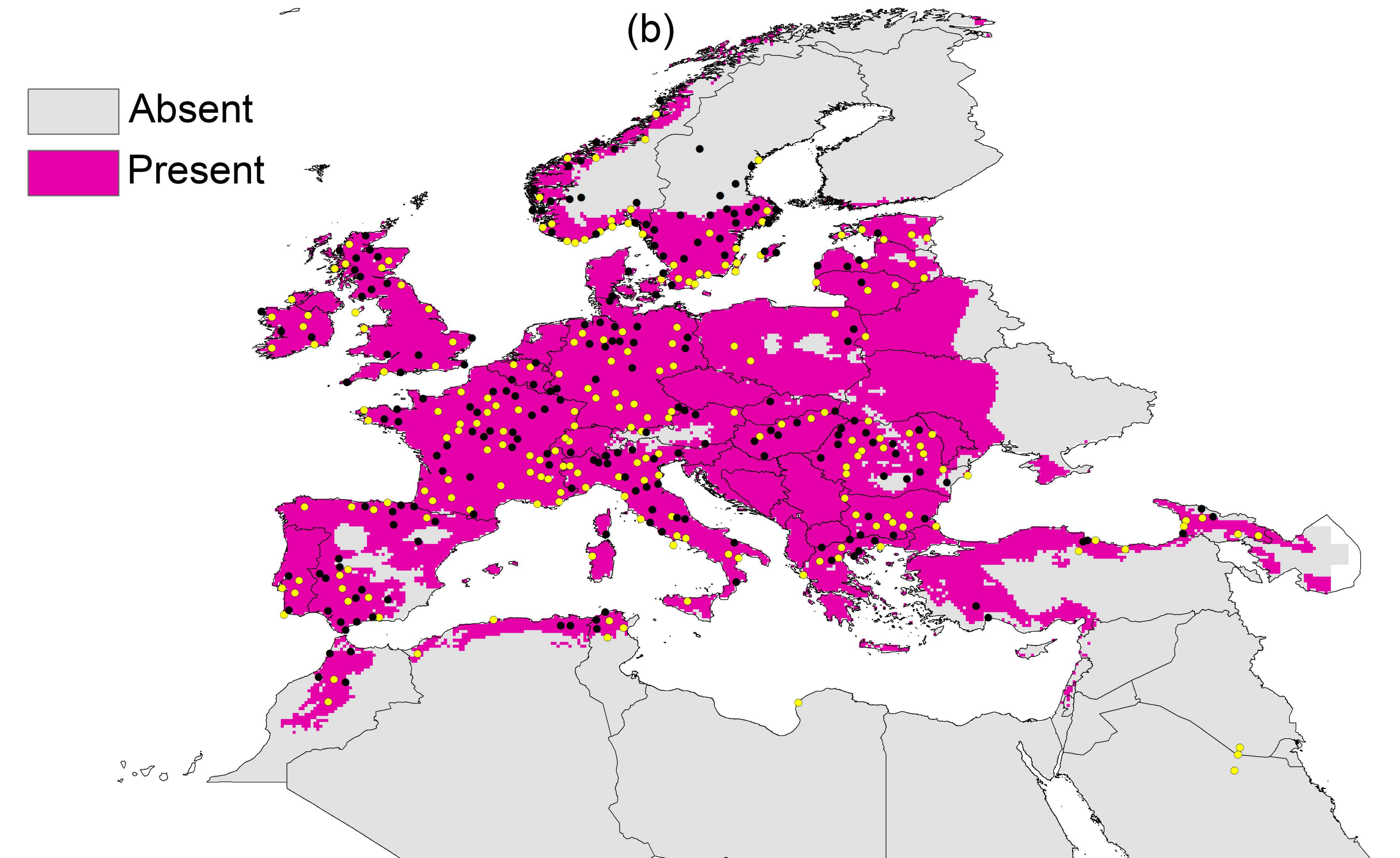
(b)
Absent
Present

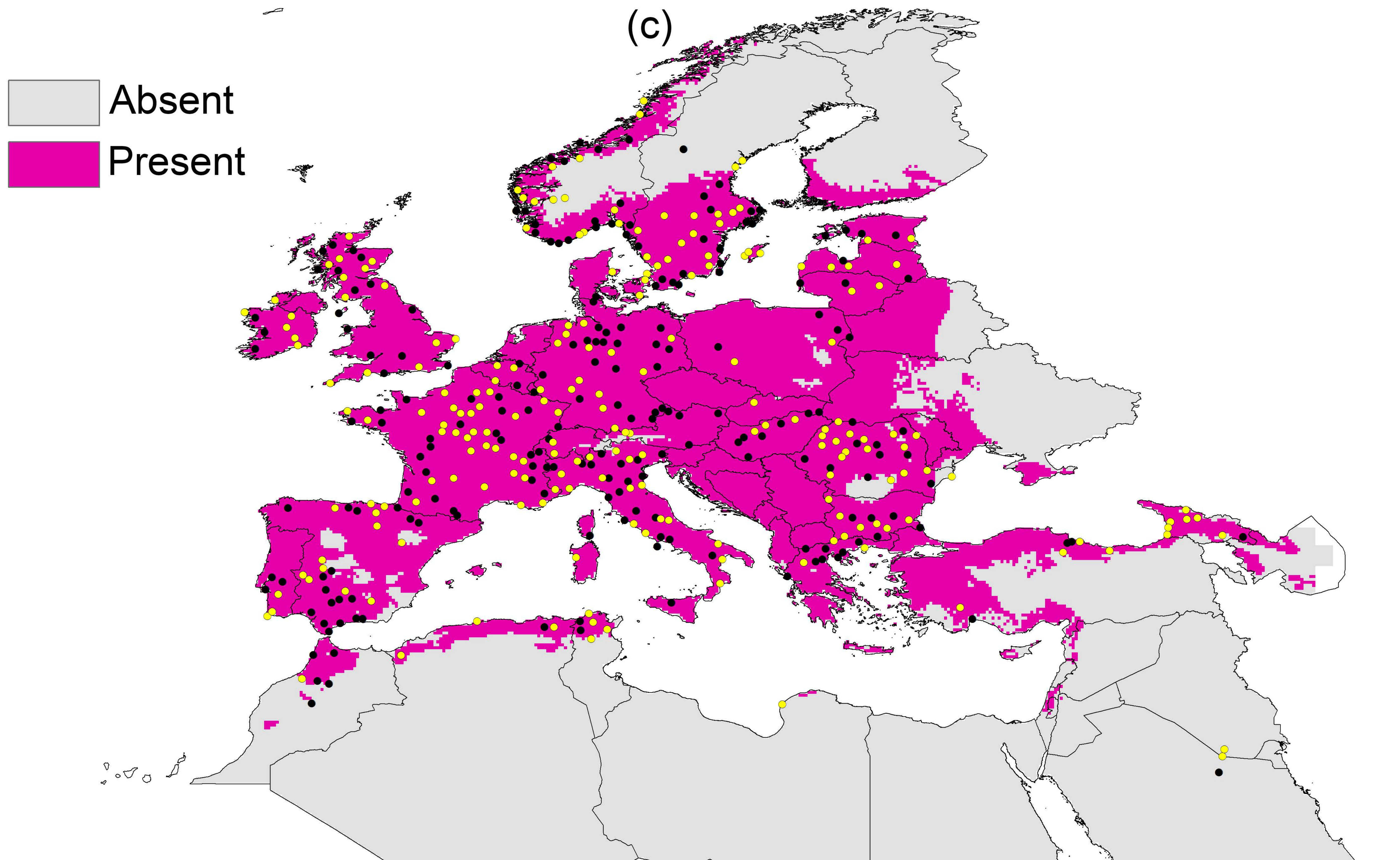
(c)
Absent
Present

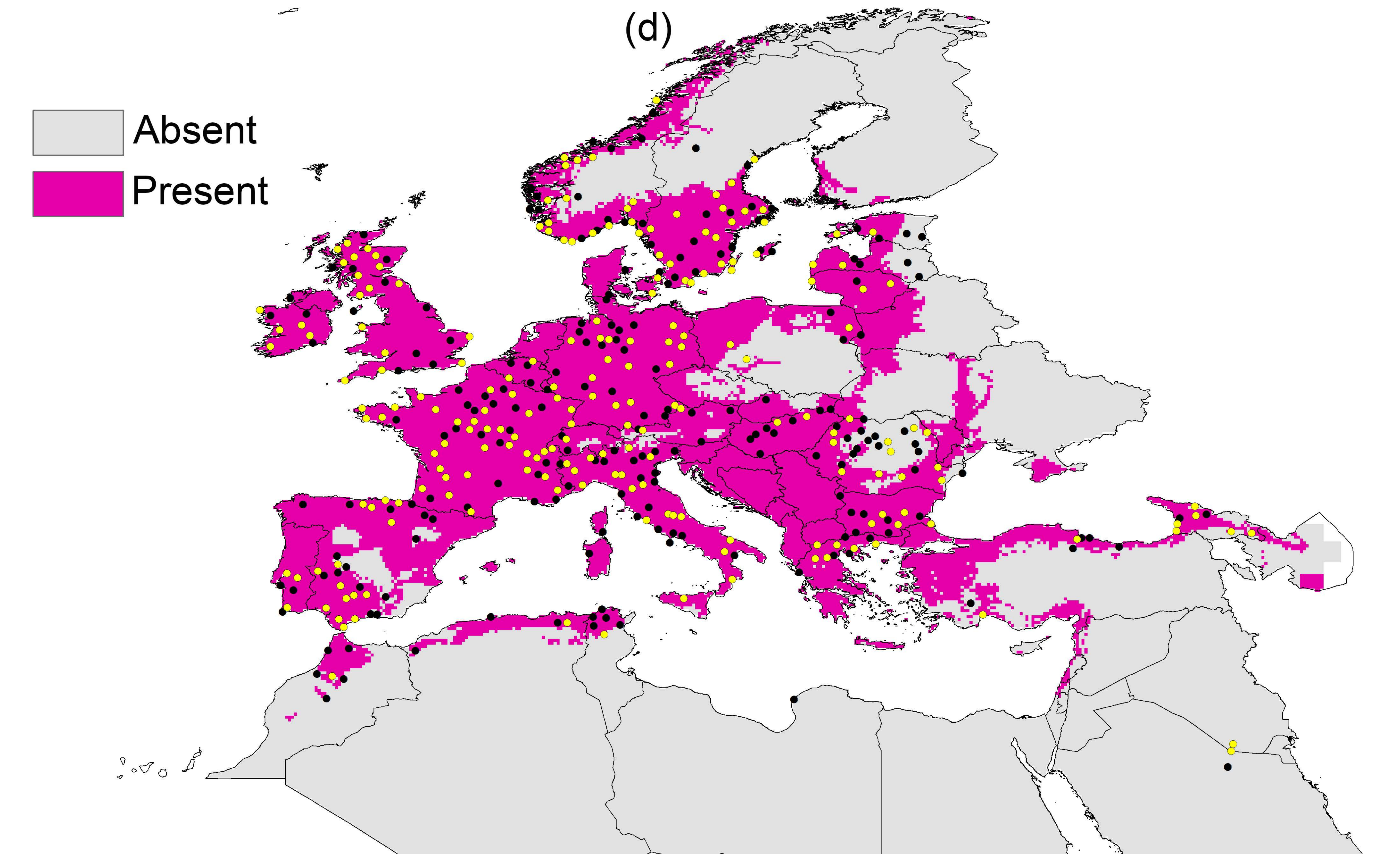
(d)
Absent
Present

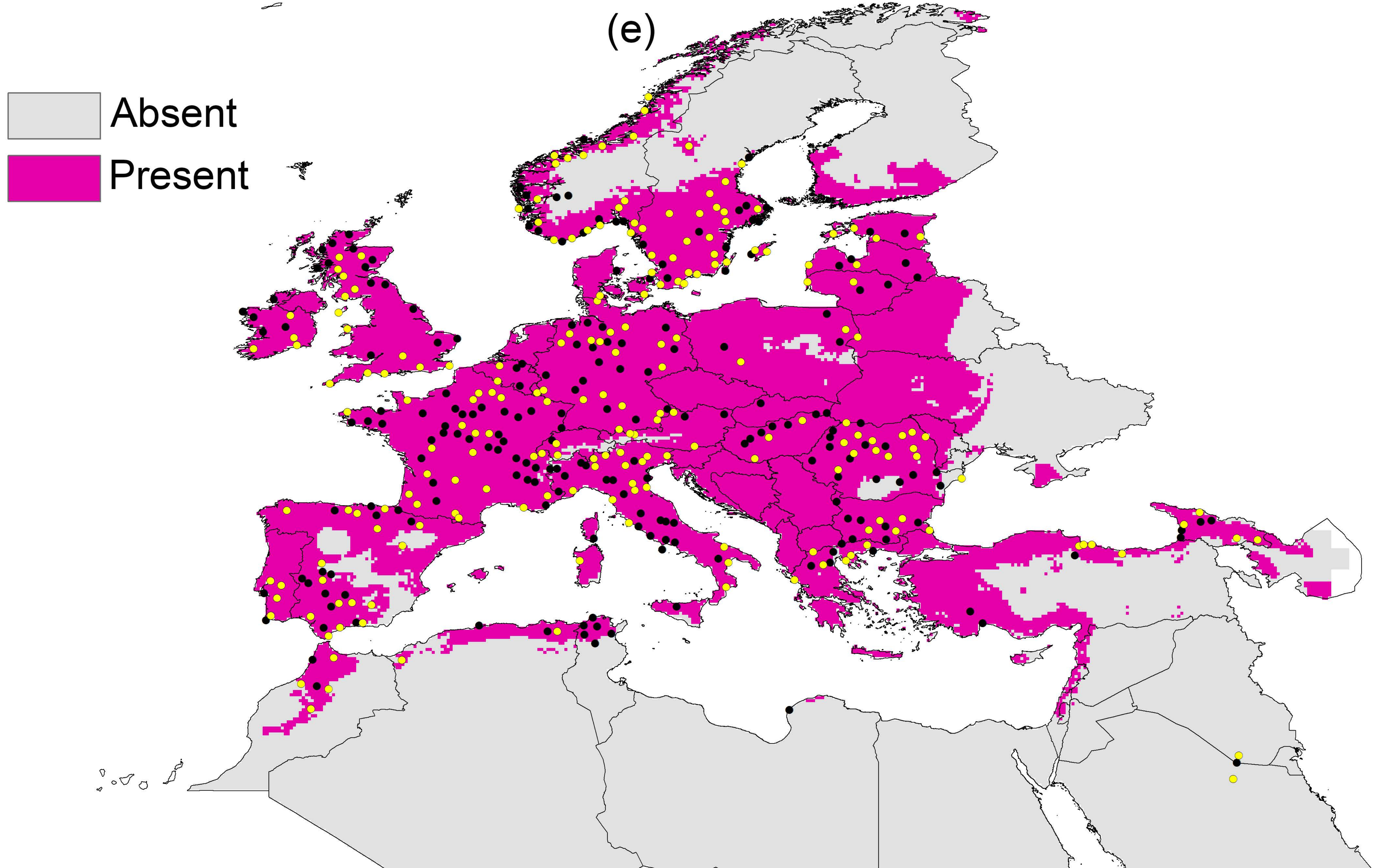
(e)
Absent
Present
