# Supplementary material for: Climate change influences on the potential geographic distribution of the disease vector tick Ixodes ricinus
Source: PLoS One. 2017 Dec 5;12(12):e0189092. doi: 10.1371/journal.pone.0189092 (PMC5716528; doi:10.1371/journal.pone.0189092)
Supplement: S3 File — The testing records are those retained from the original occurrences (i.e. black dots) but none coincided with the 416 records used in model calibration (i.e. yellow circles). (PDF) [file pone.0189092.s003.pdf]

S3 File. Relationship of ecological niche model predictions to the distribution of 416 and 3186 records of *Ixodes ricinus* occurrences used for model calibration and testing, respectively. The testing records are those retained from the original occurrences (i.e. black dots) but none coincided with the 416 records used in model calibration (i.e. yellow circles).

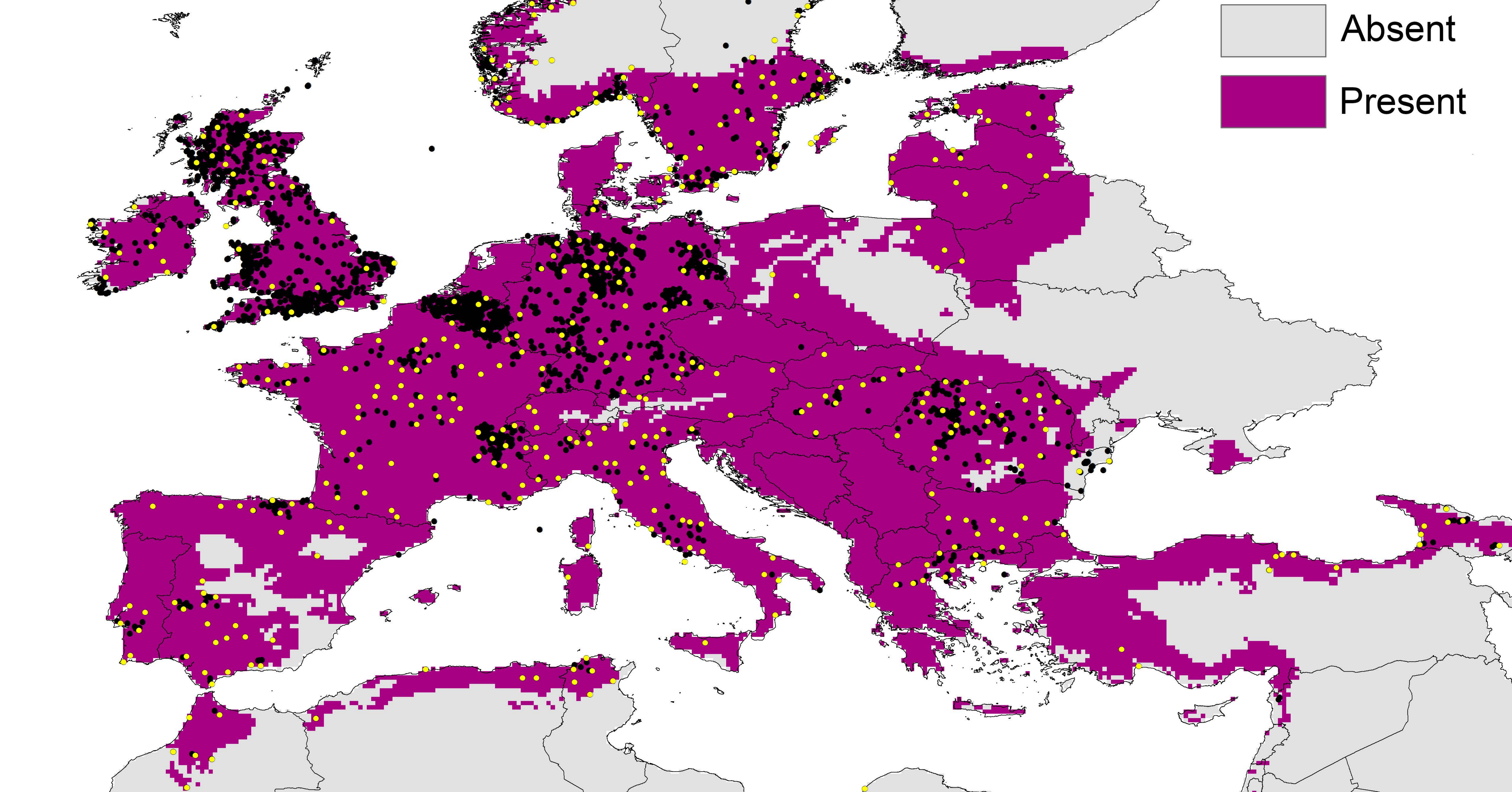
Absent
Present
