# Supplementary material for: Climate change influences on the potential geographic distribution of the disease vector tick Ixodes ricinus
Source: PLoS One. 2017 Dec 5;12(12):e0189092. doi: 10.1371/journal.pone.0189092 (PMC5716528; doi:10.1371/journal.pone.0189092)
Supplement: S4 File — This supporting information shows the results regarding the relative contributions of six environmental variables to models for I. ricinus based on 5 random subsets of occurrences. (PDF) [file pone.0189092.s004.pdf]

S4 File. Relative contributions of six environmental variables to the ecological niche models for *Ixodes ricinus* and its response to those variables. This supporting information shows the results regarding the relative contributions of six environmental variables to models for *I. ricinus* based on 5 random subsets of occurrences.

Table I. The relative contributions of the environmental variables to the ecological niche models for *Ixodes ricinus* based on five random subsets selected in the analyses.

**Variable**	**% Contribution**				
	**Subset #1**	**Subset #2**	**Subset #3**	**Subset #4**	**Subset #5**
**Annual Precipitation**	56.5	55.1	50.3	55	57.3
**Temperature Annual Range**	21.5	21.8	28.1	23.8	25.1
**Annual Mean Temperature**	8.1	11.3	10.6	8.6	11.1
**Precipitation Seasonality**	5.8	2.8	5.2	7.4	1.6
**Isothermality**	4.6	3.6	3.3	2.9	1.5
**Mean Diurnal Range**	3.5	5.3	2.6	2.2	3.4

Figure I. Response curves of *Ixodes ricinus* to the six environmental variables included in the model based on five random subsets of occurrences; a) subset #1, b) subset #2, c) subset #3, d) subset #4, and e) subset #5. The graph shows how the Maxent logistic prediction (y-axis) varies across the range of values of each predictor when used in a one-variable model. Red line presents the mean response of 10 iterations of the Maxent runs and the blue area presents mean ± one standard deviation.

(a)

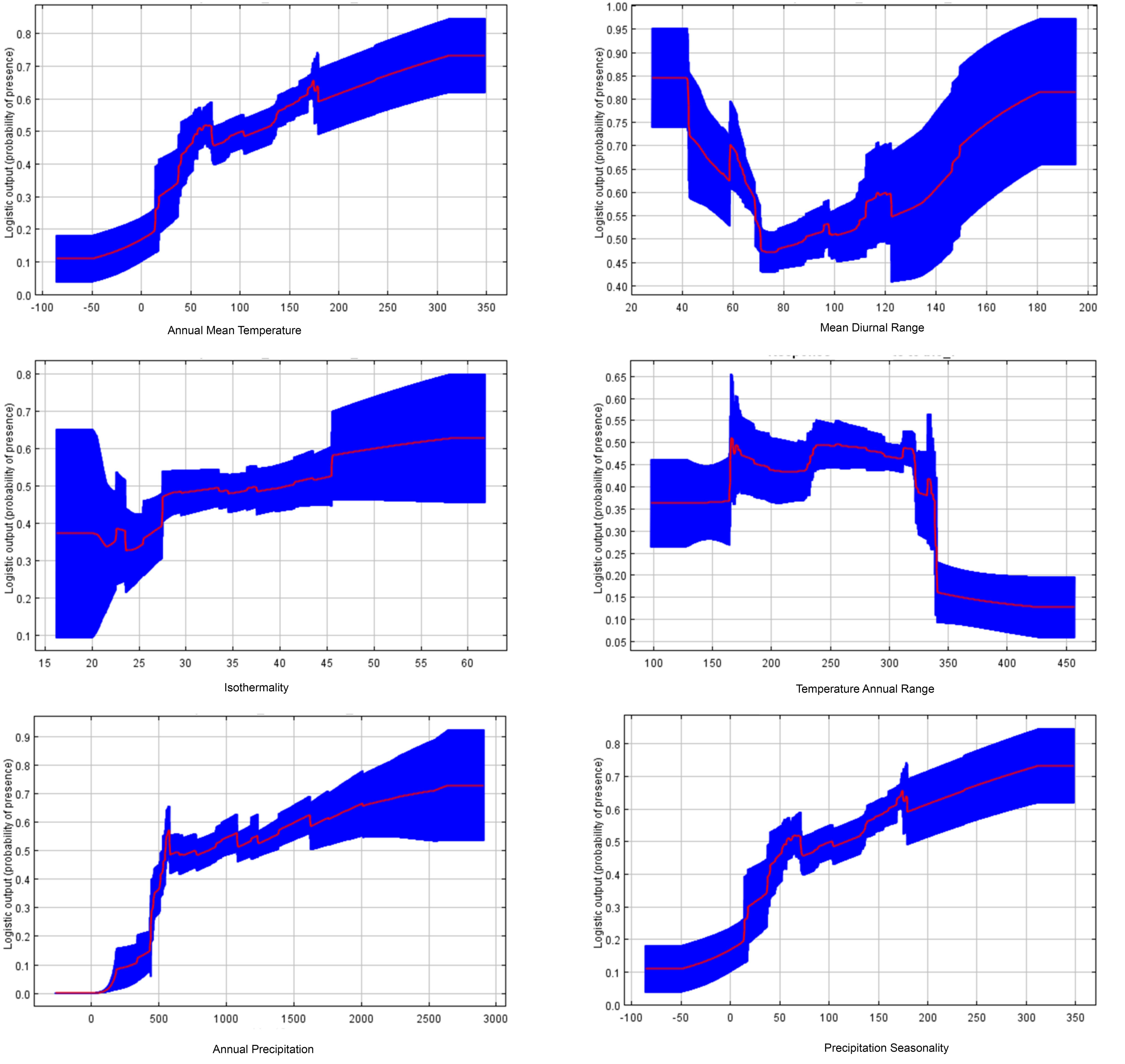
Logistic output (probability of presence)
Annual Mean Temperature
Mean Diurnal Range
Isothermality
Temperature Annual Range
Annual Precipitation
Precipitation Seasonality

(b)

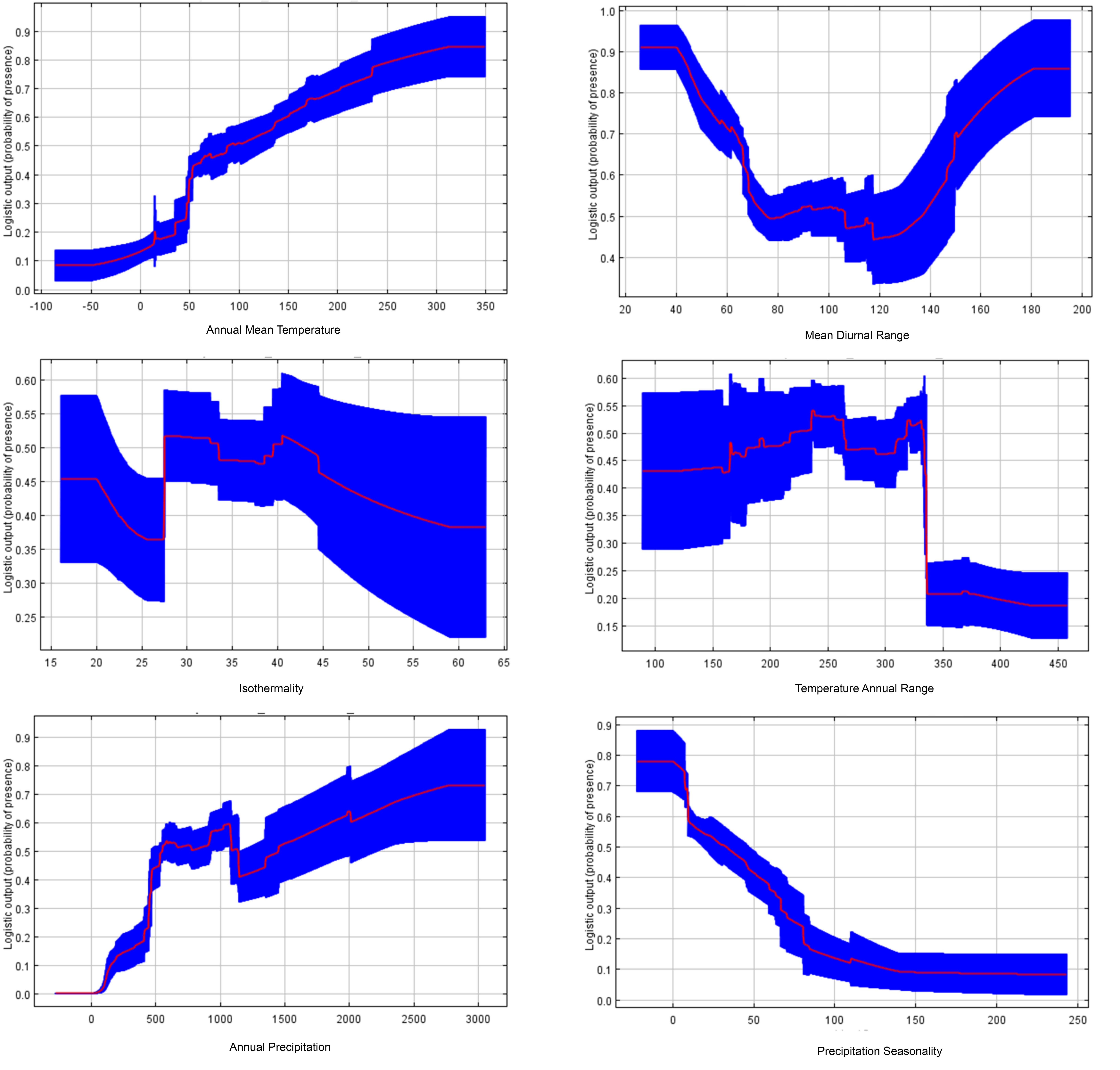

Logistic output (probability of presence)
Annual Mean Temperature
Logistic output (probability of presence)
Mean Diurnal Range
Logistic output (probability of presence)
Isothermality
Logistic output (probability of presence)
Temperature Annual Range
Logistic output (probability of presence)
Annual Precipitation
Logistic output (probability of presence)
Precipitation Seasonality

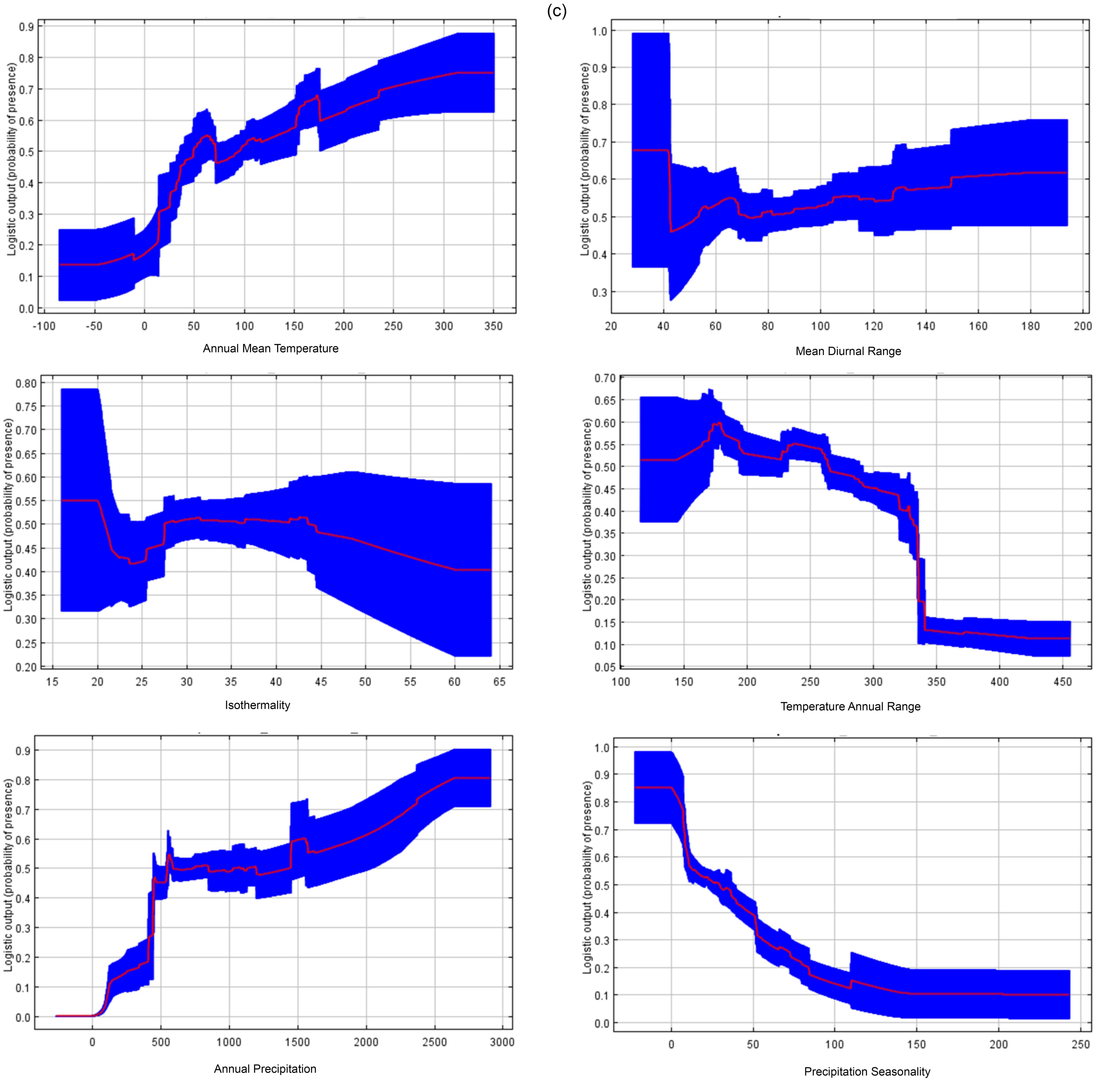
(c)
Logistic output (probability of presence)
Annual Mean Temperature
Mean Diurnal Range
Isothermality
Temperature Annual Range
Annual Precipitation
Precipitation Seasonality

(d)

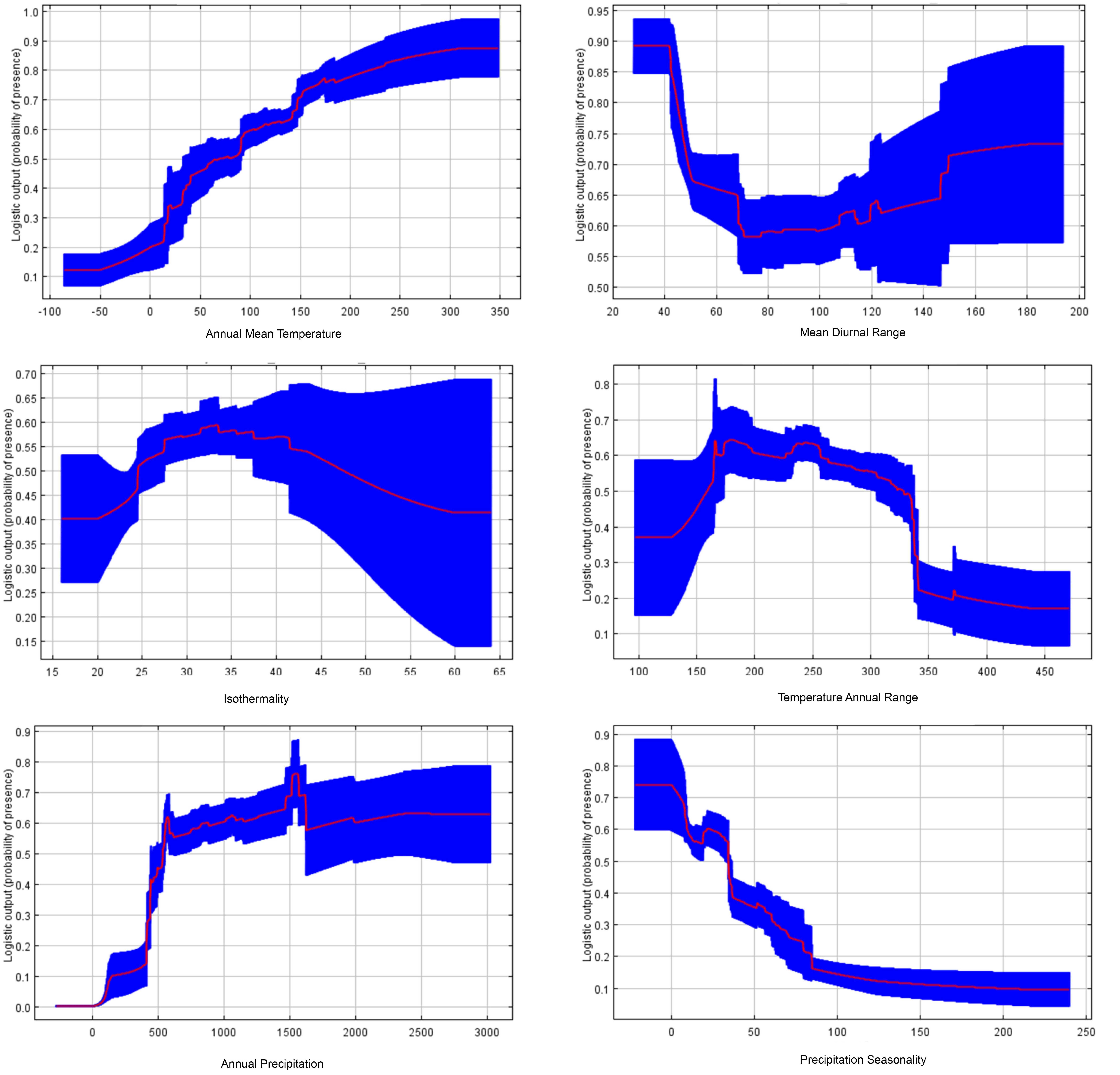
Logistic output (probability of presence)
Annual Mean Temperature
Mean Diurnal Range
Isothermality
Temperature Annual Range
Annual Precipitation
Precipitation Seasonality

(e)

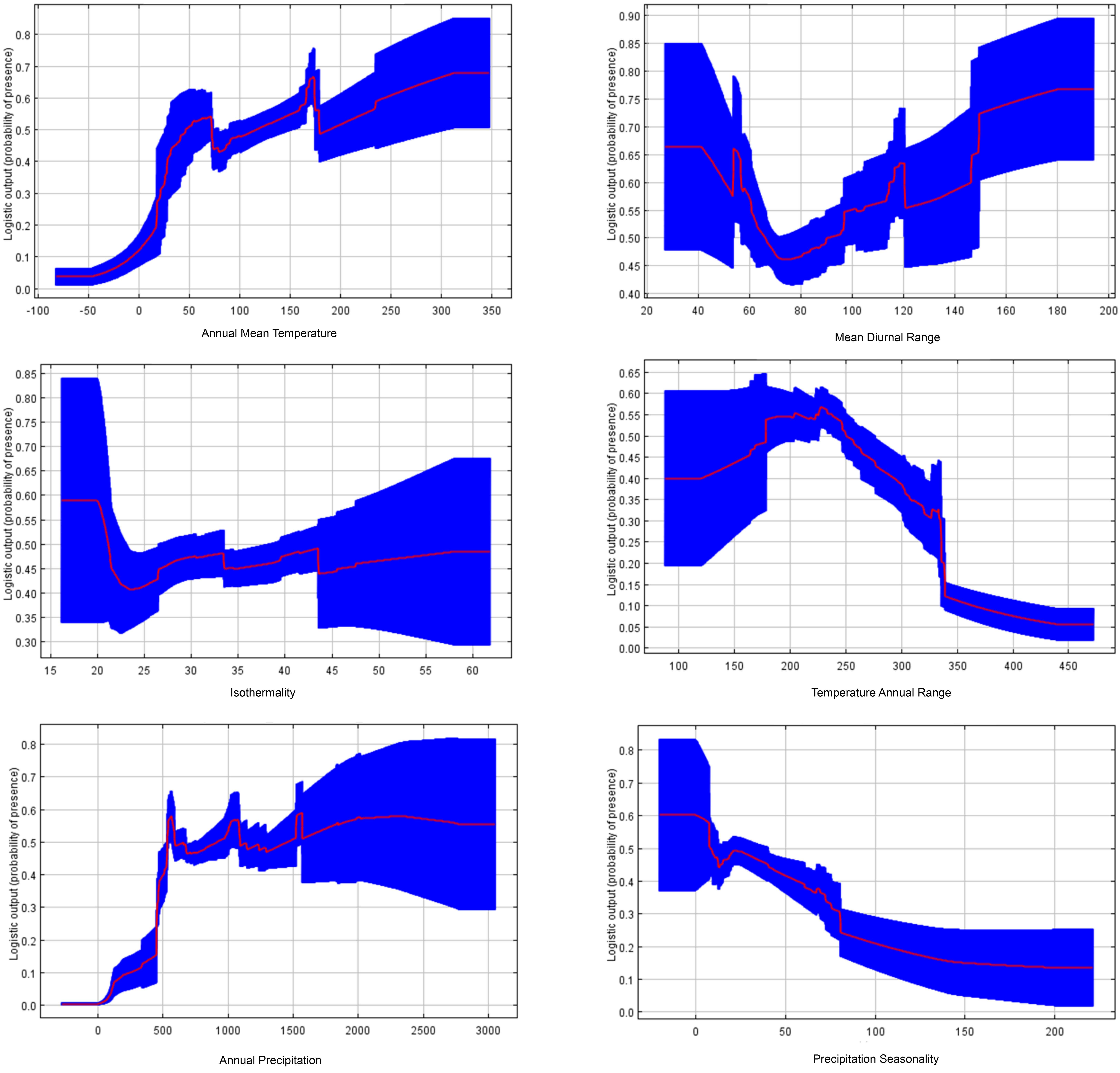

Logistic output (probability of presence)
Annual Mean Temperature
Logistic output (probability of presence)
Mean Diurnal Range
Logistic output (probability of presence)
Isothermality
Logistic output (probability of presence)
Temperature Annual Range
Logistic output (probability of presence)
Annual Precipitation
Logistic output (probability of presence)
Precipitation Seasonality
