# Supplementary material for: Climate change influences on the potential geographic distribution of the disease vector tick Ixodes ricinus
Source: PLoS One. 2017 Dec 5;12(12):e0189092. doi: 10.1371/journal.pone.0189092 (PMC5716528; doi:10.1371/journal.pone.0189092)

Supplementary File 2 (S2 File)

This supplementary file describes the predicted potential distribution of *Ixodes ricinus* based on each individual General Circulation Model (GCM). Each individual GCM is presented in two representative concentration pathways presenting different emission in two periods; 2050, and 2070. The color scheme on the map presented the stability in model prediction based on both current and future conditions; Dark blue indicates present and future suitable prediction, light blue indicates present suitable prediction, and red indicates future suitable prediction.

1. The predicted potential distribution based on the GCM of the Commonwealth Scientific and Industrial Research Organization (CSIRO) and Bureau of Meteorology (BOM), Australia (ACCESS 1 – 0).

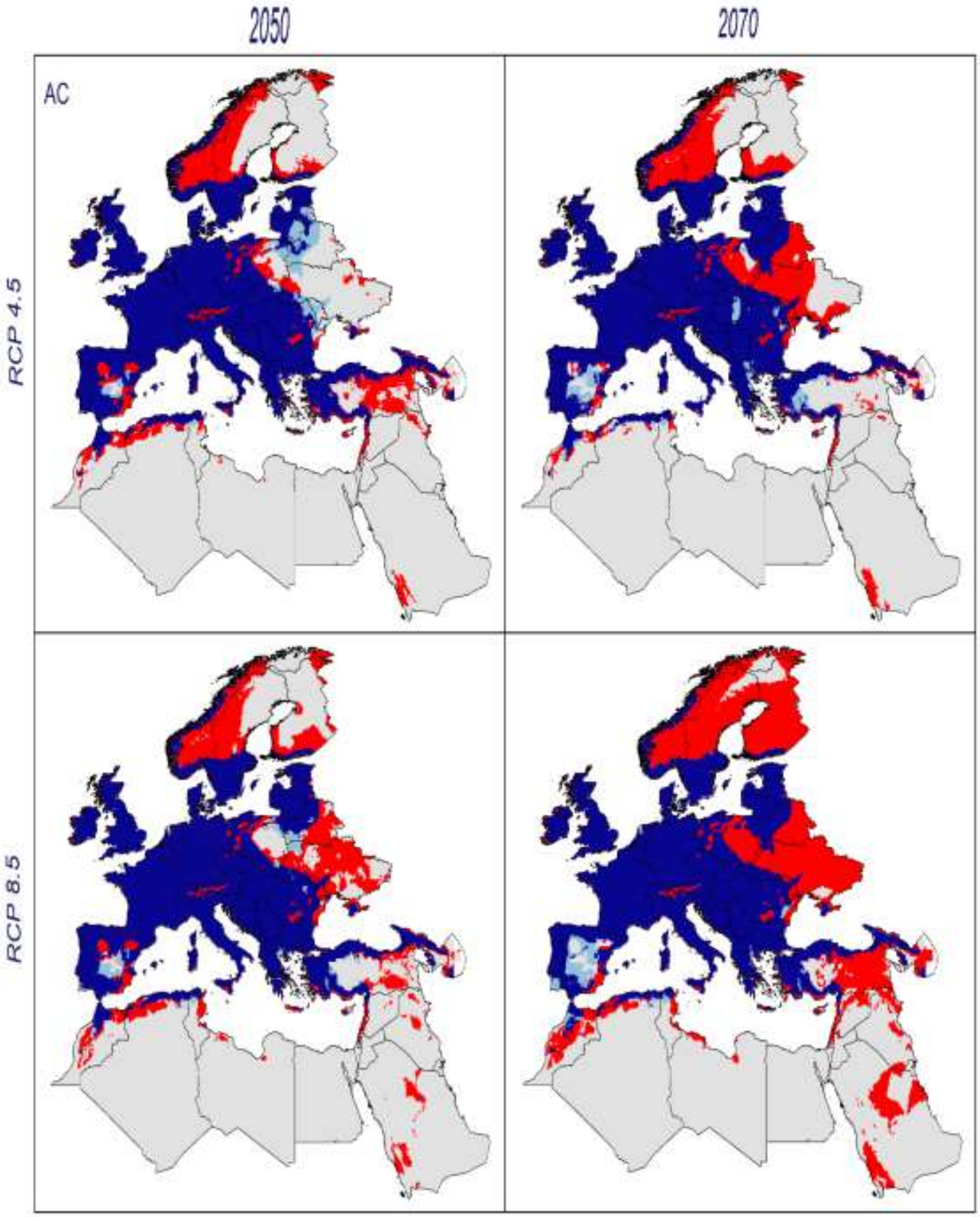

2. The predicted potential distribution based on the GCM of the Beijing Climate Center, China Meteorological Administration, China (BCC – CSM 1 – 1).

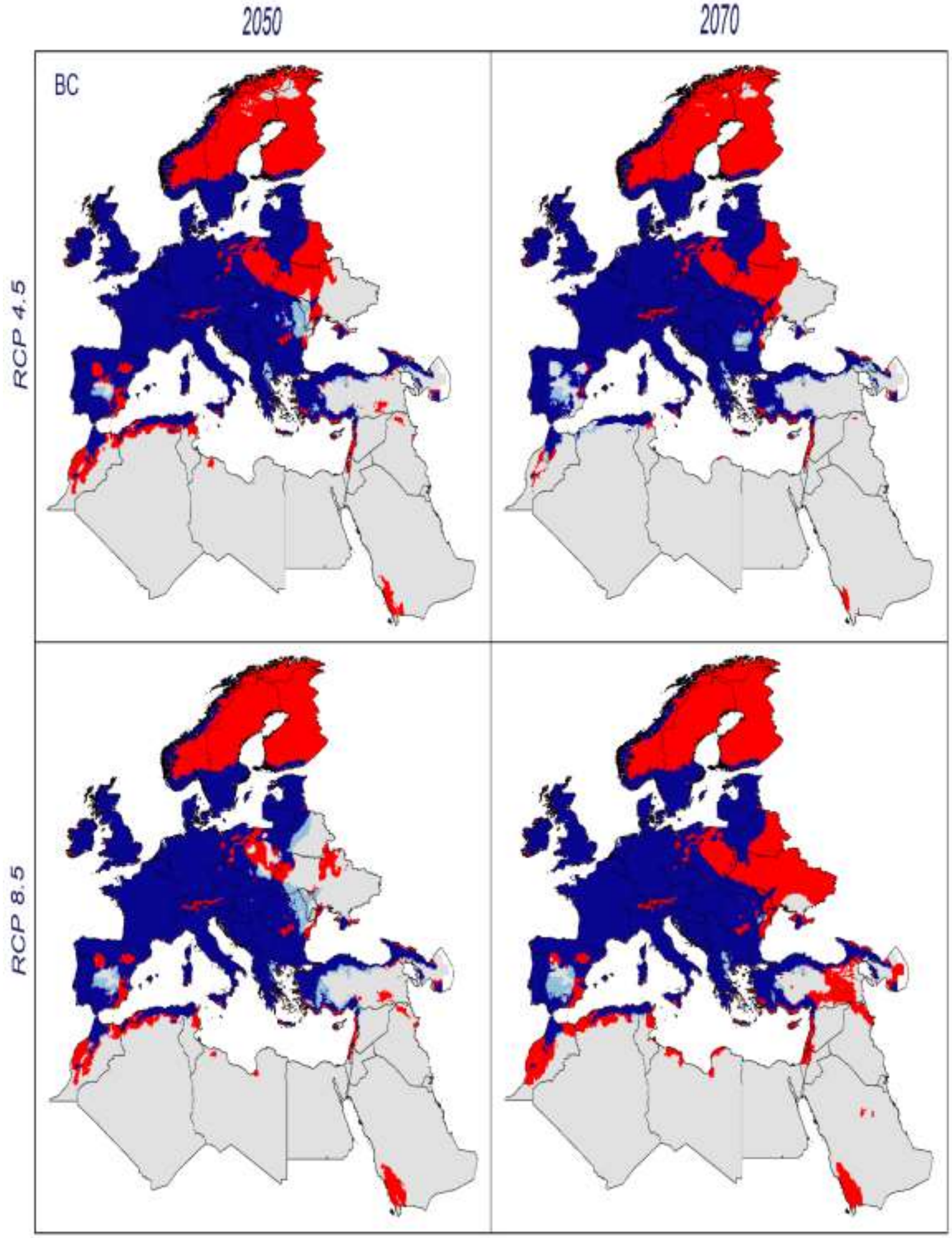

3. The predicted potential distribution based on the GCM of the National Center for Atmospheric Research, USA (CCSM 4).

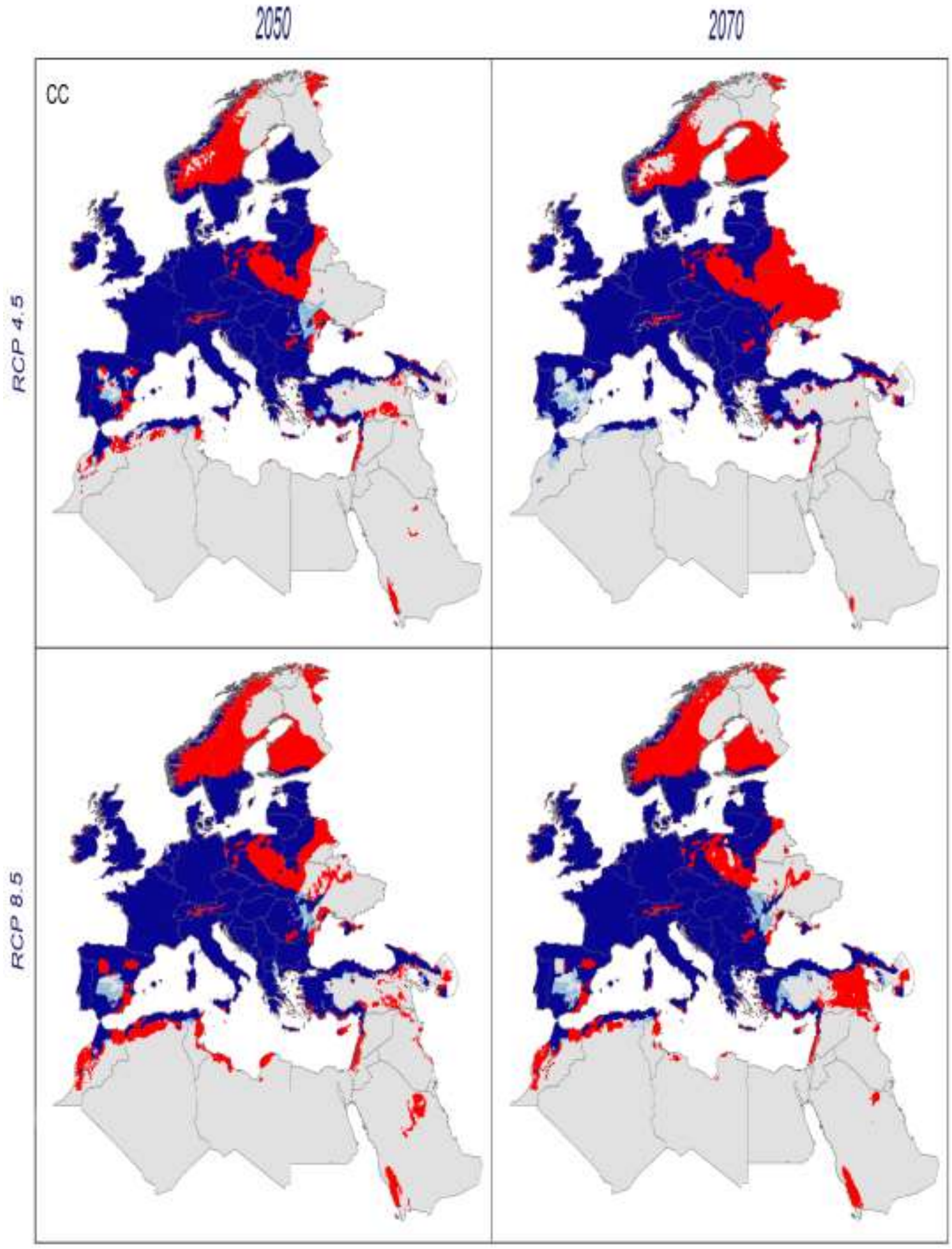

4. The predicted potential distribution based on the GCM of the Centre National de Recherches Météorologiques, France (CNRM – CM 5).

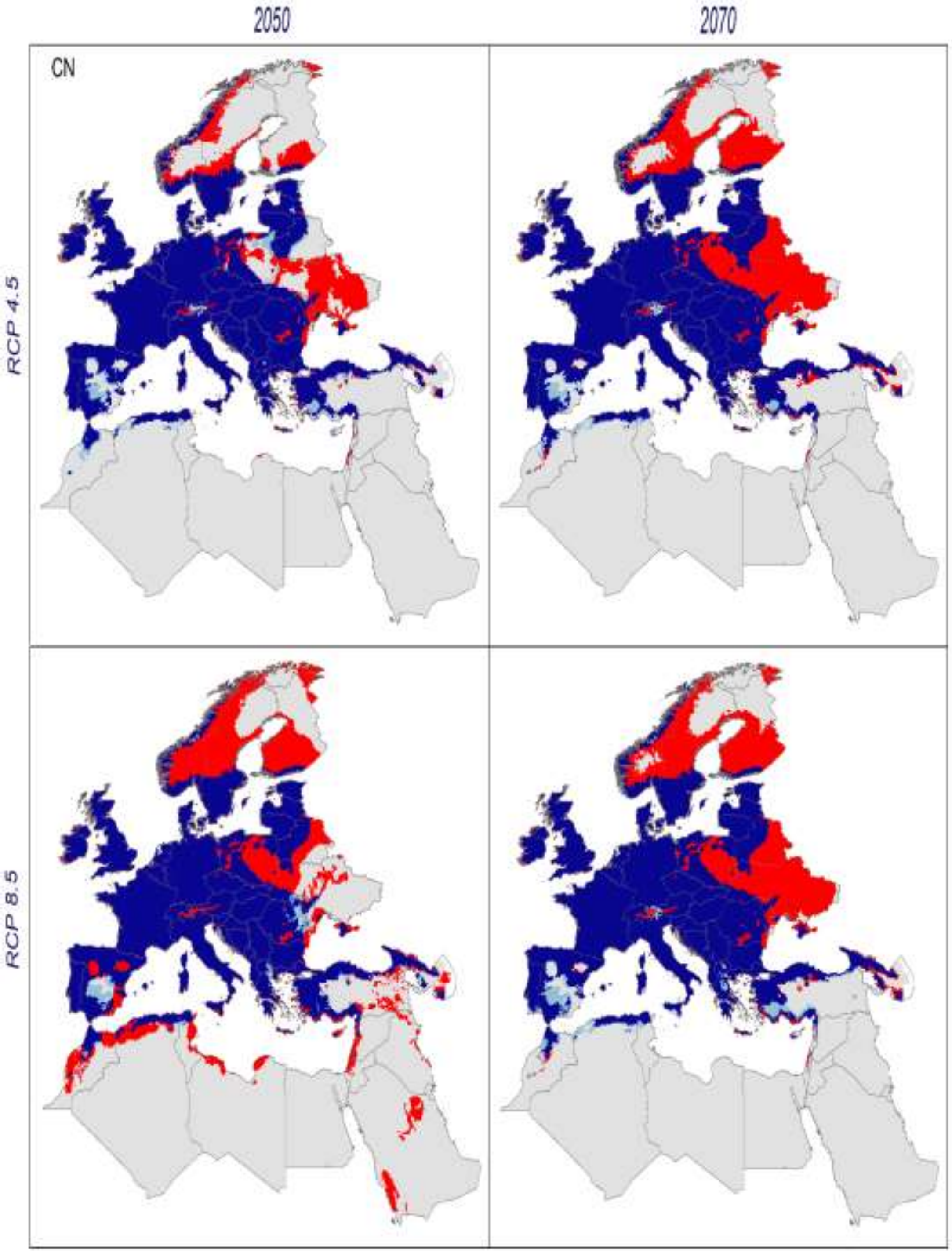

5. The predicted potential distribution based on the GCM of the NOAA Geophysical Fluid Dynamics Laboratory, USA (GFDL – CM 3).

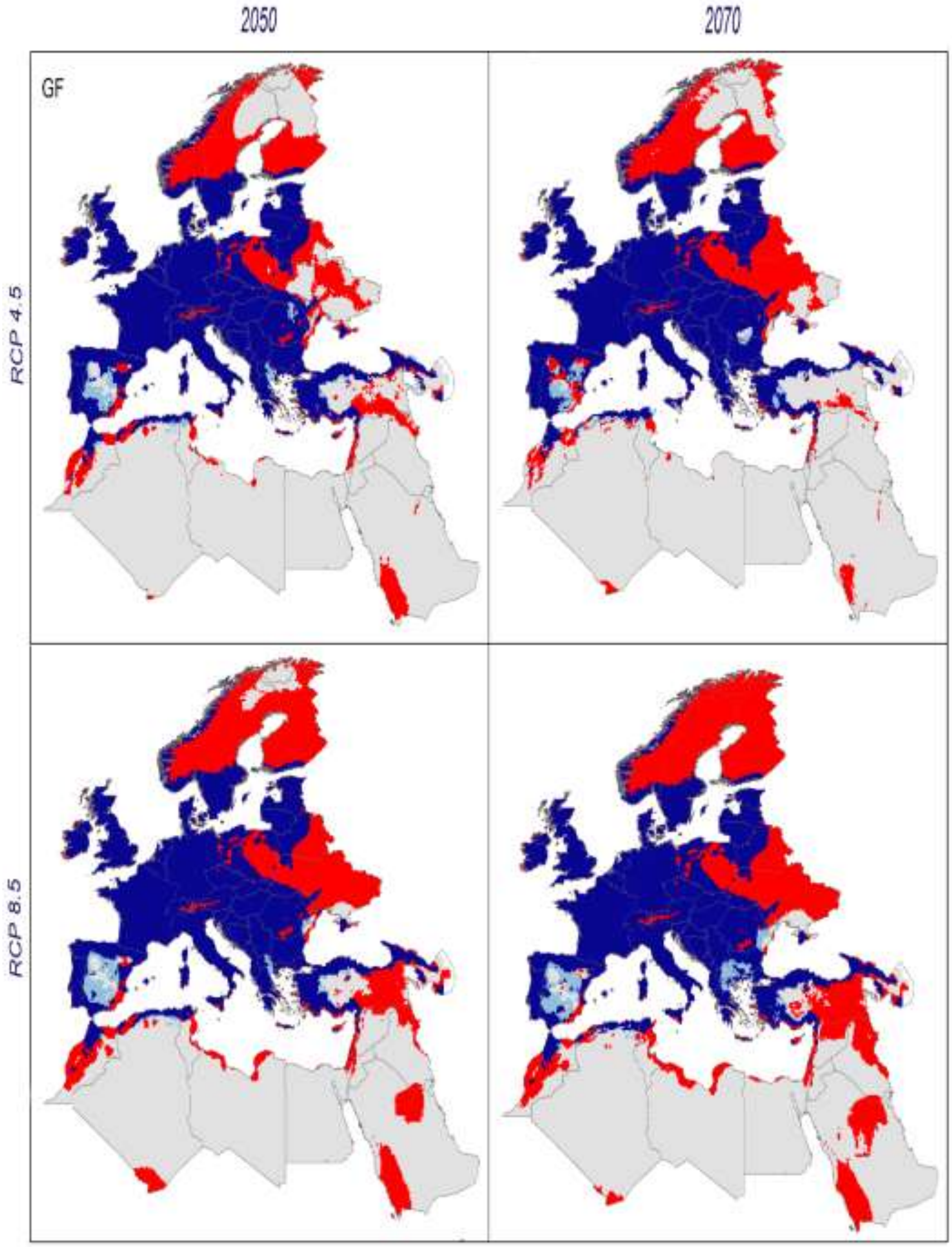

6. The predicted potential distribution based on the GCM of the NASA Goddard Institute for Space Studies, USA (GISS – E2 - R).

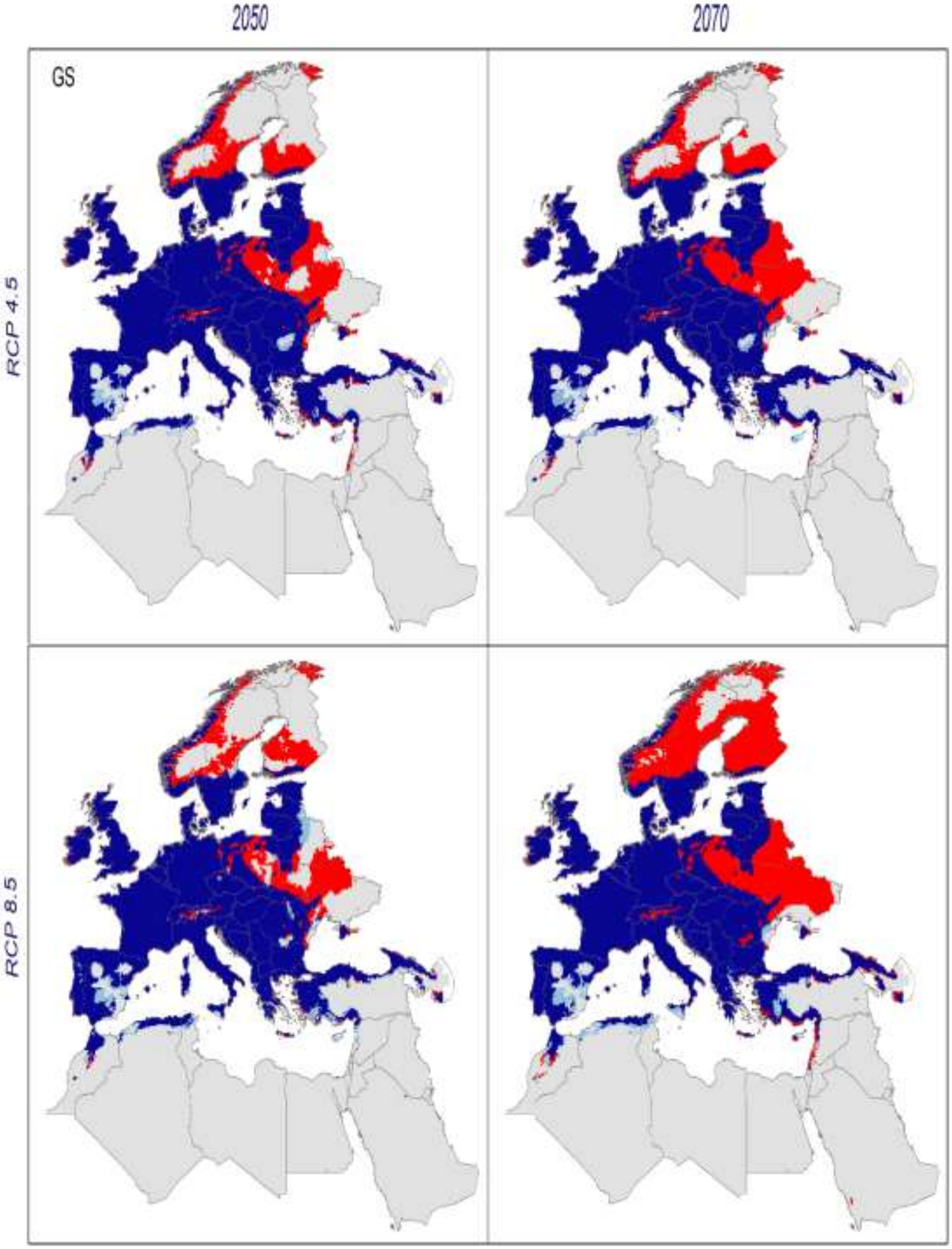

7. The predicted potential distribution based on the GCM of the National Institute of Meteorological Research, Korea Meteorological Administration, Korea (HadGEM 2 – AO).

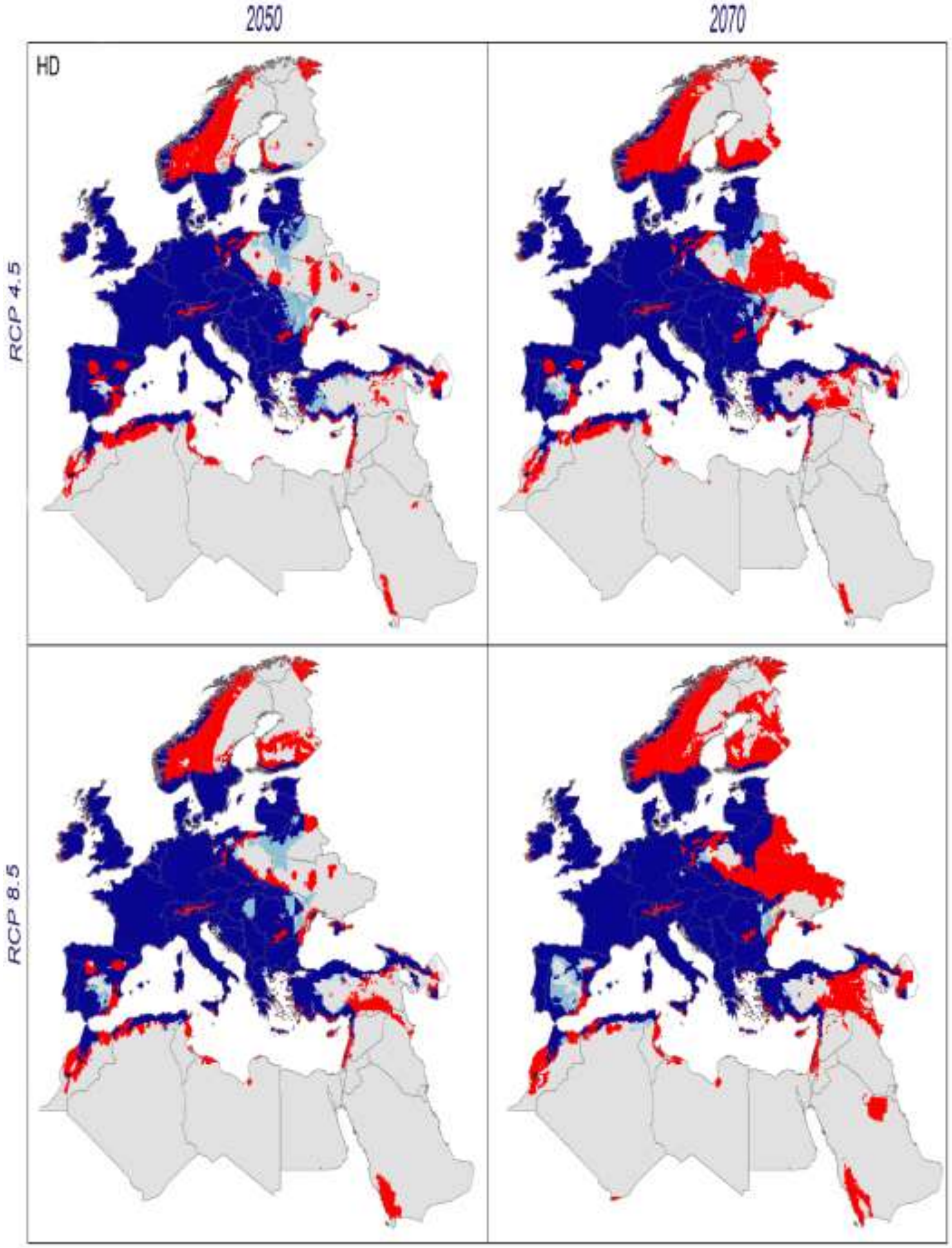

8. The predicted potential distribution based on the GCM of the Met Office Hadley Centre (additional HadGEM2-ES realizations contributed by Instituto Nacional de Pesquisas Espaciais) (HadGEM 2-ES).

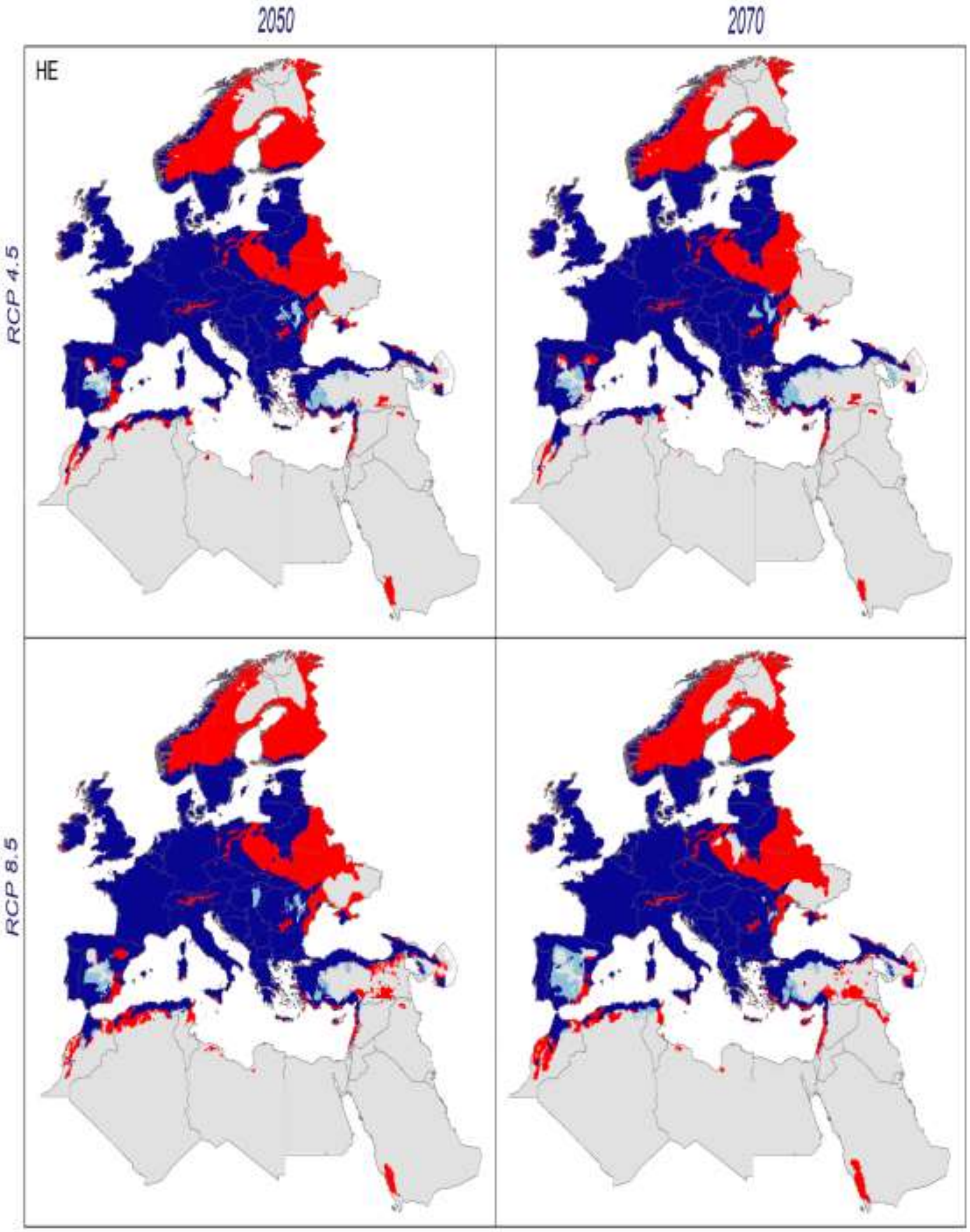

9. The predicted potential distribution based on the GCM of the Met Office Hadley Centre (additional HadGEM2-ES realizations contributed by Instituto Nacional de Pesquisas Espaciais) (HadGEM 2 – CC).

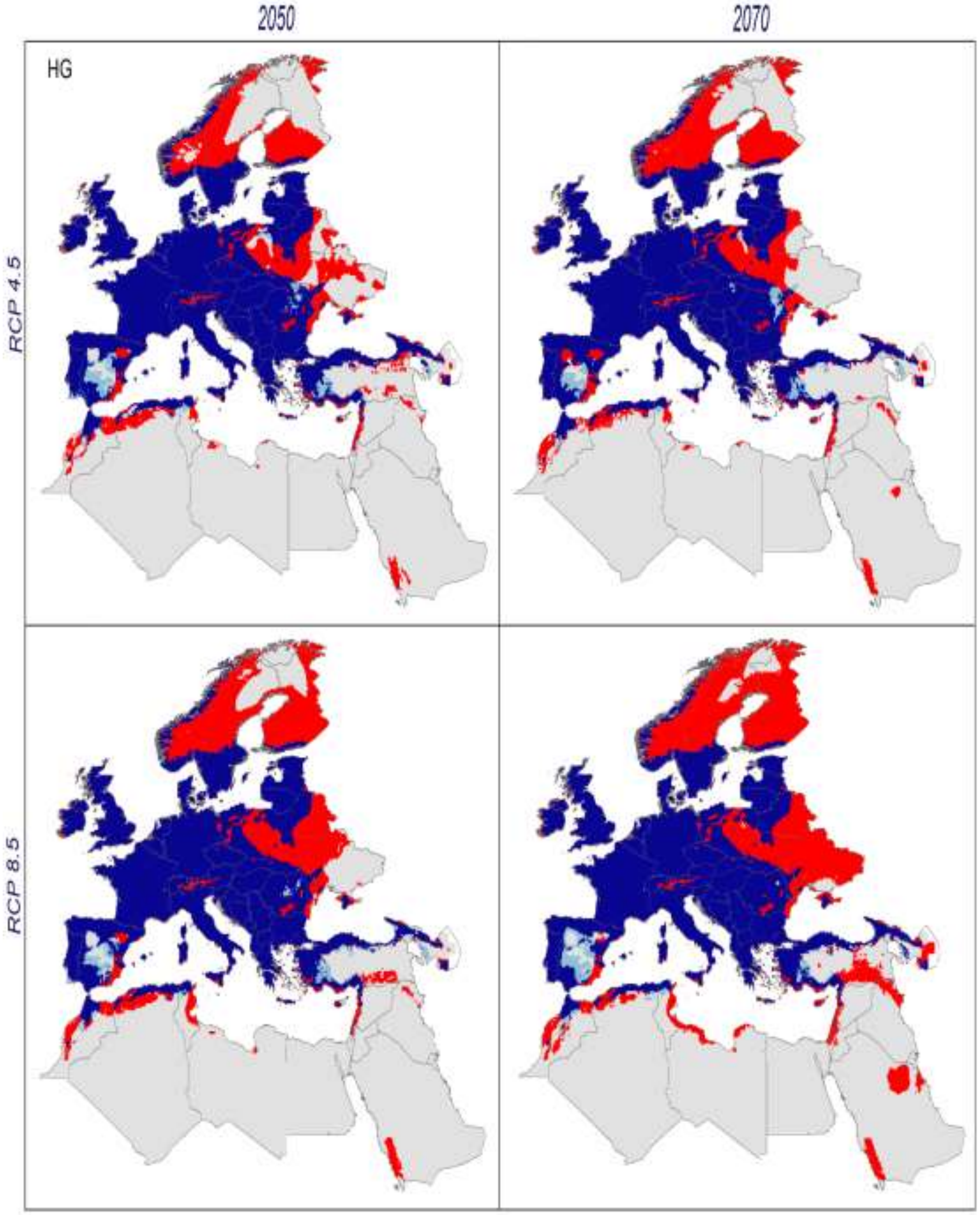

10. The predicted potential distribution based on the GCM of the Institute for Numerical Mathematics, Russia (INMCM 4).

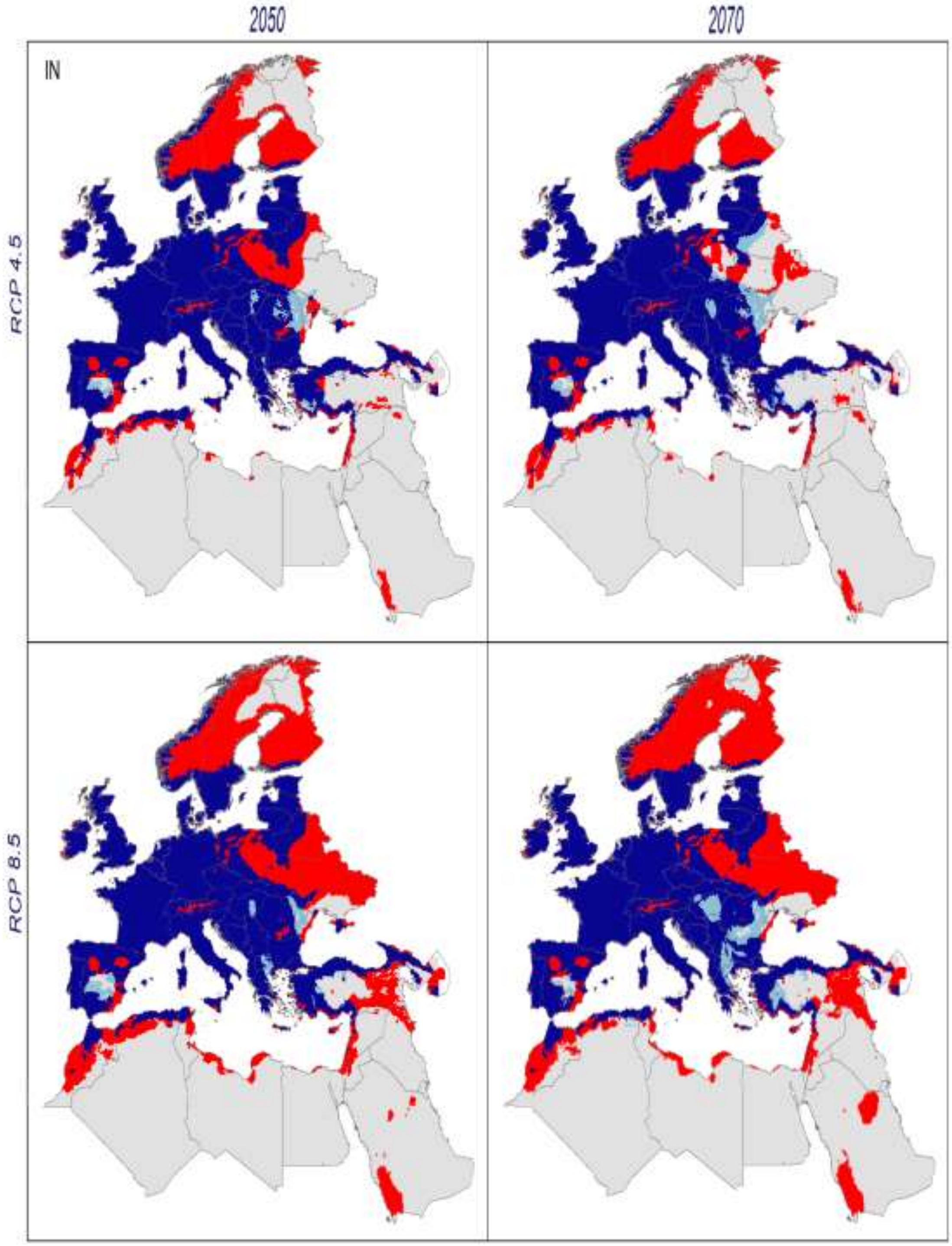

11. The predicted potential distribution based on the GCM of the Institute Pierre-Simon Laplace, France (IPSL – CM5A – LR).

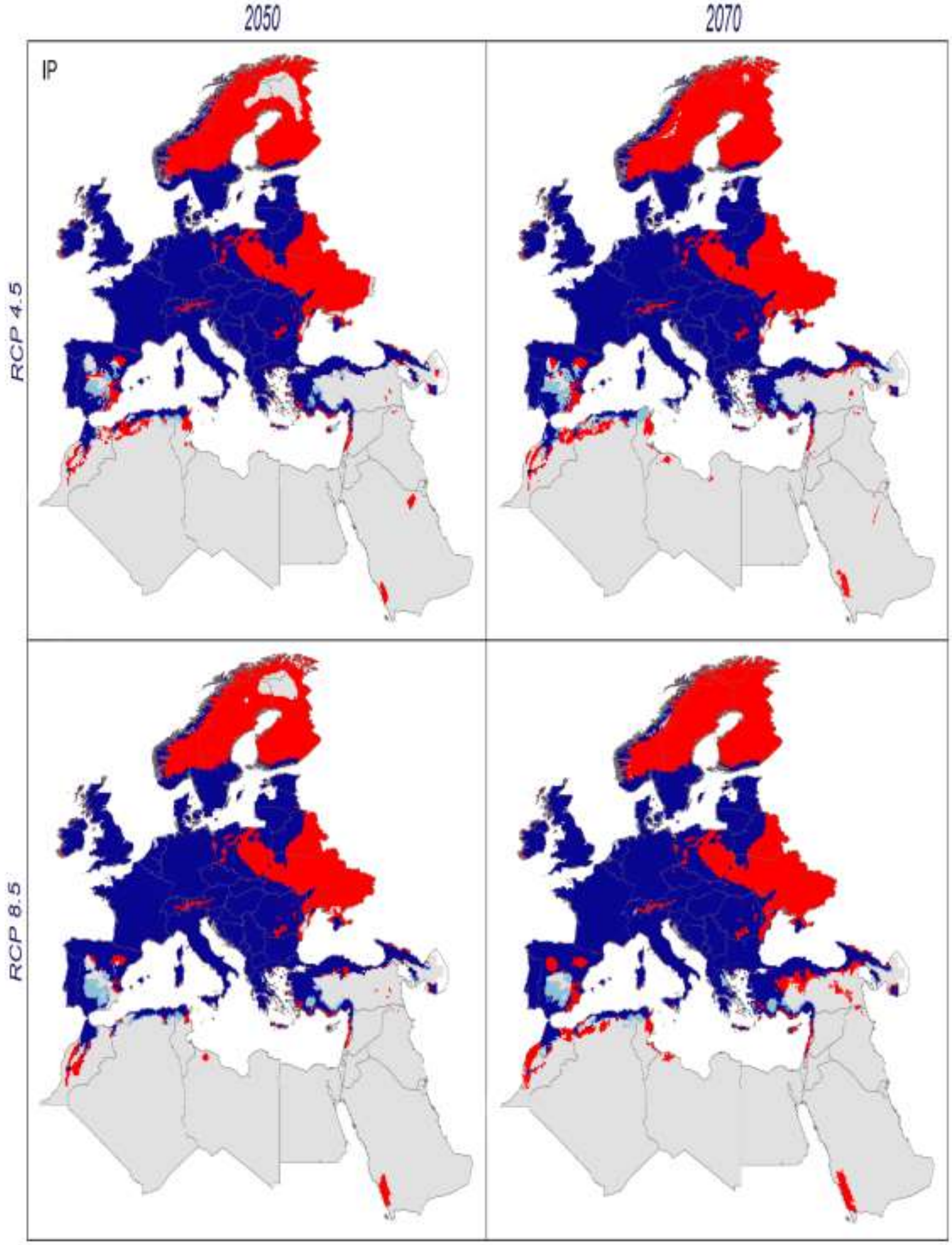

12. The predicted potential distribution based on the GCM of the Atmosphere and Ocean Research Institute (The University of Tokyo), National Institute for Environmental Studies, and Japan Agency for Marine-Earth Science and Technology, Japan (MIROC 5).

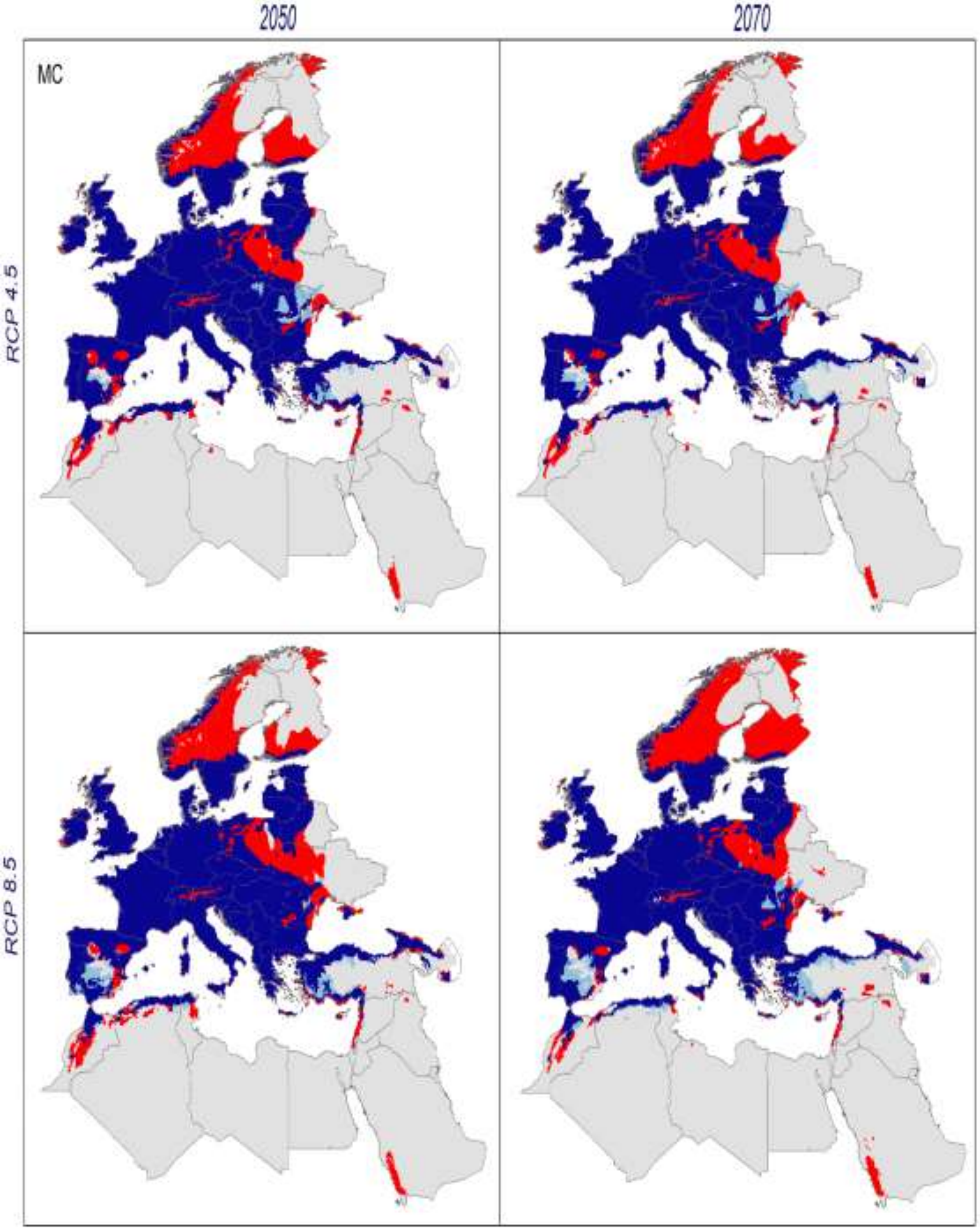

13. The predicted potential distribution based on the GCM of the Meteorological Research Institute, Japan (MRI – CGCM 3).

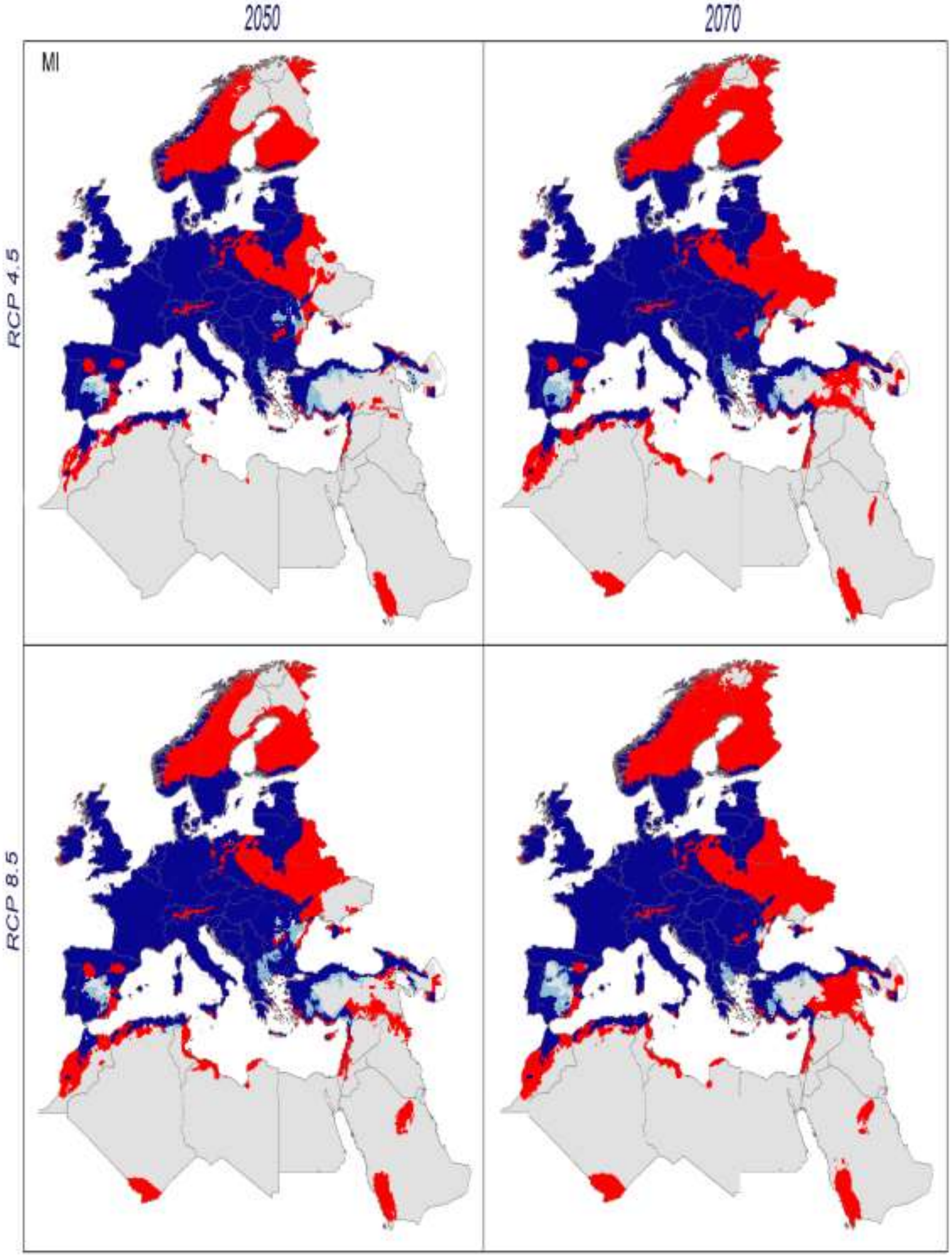

14. The predicted potential distribution based on the GCM of the Japan Agency for Marine-Earth Science and Technology, Atmosphere and Ocean Research Institute (The University of Tokyo), and National Institute for Environmental Studies, Japan (MIROC – ESM – CHEM).

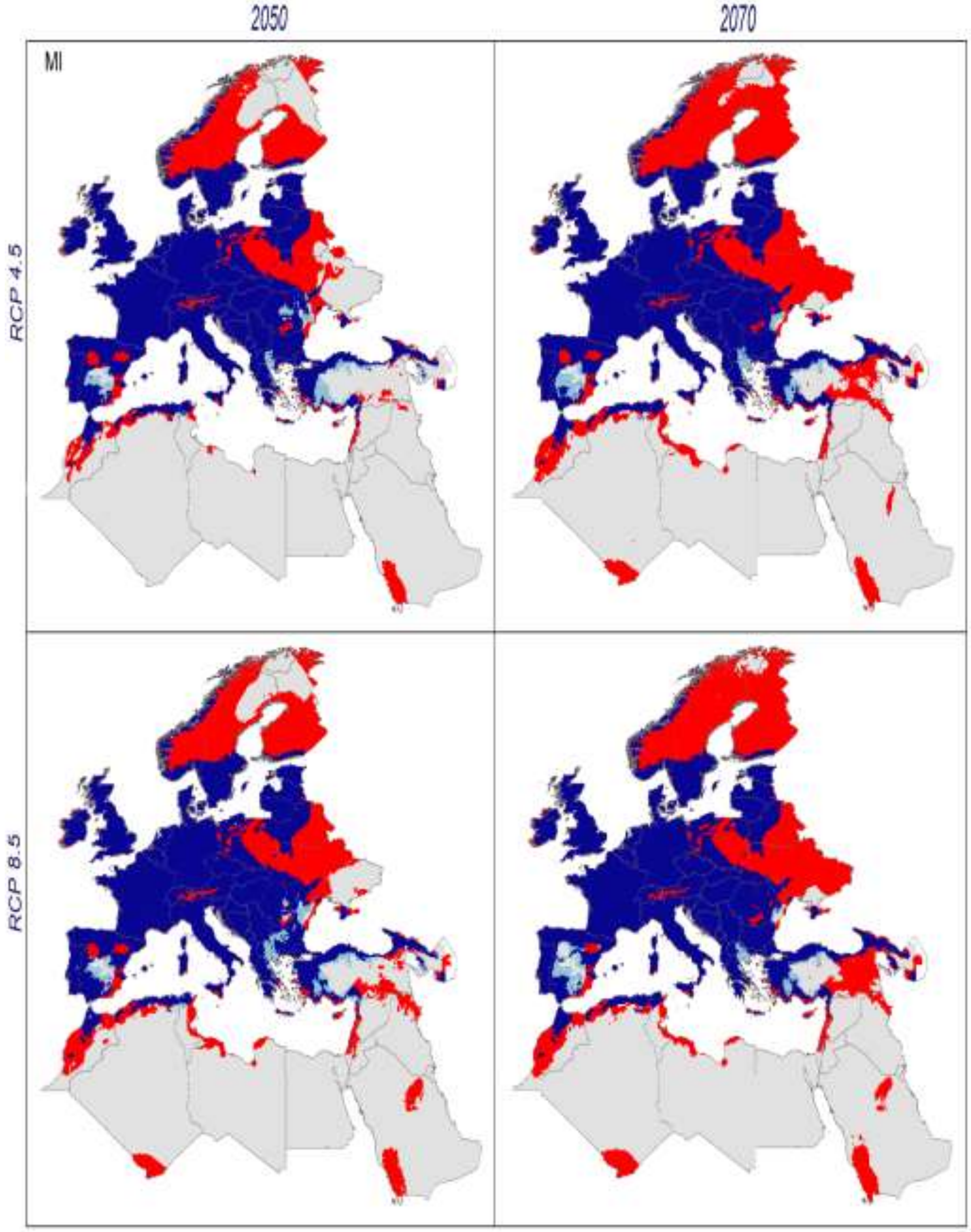

15. The predicted potential distribution based on the GCM of the Max-Planck-Institut für Meteorolgie (Max Planck Institute for Meteorology), Germany (MPI – ESM – LR).

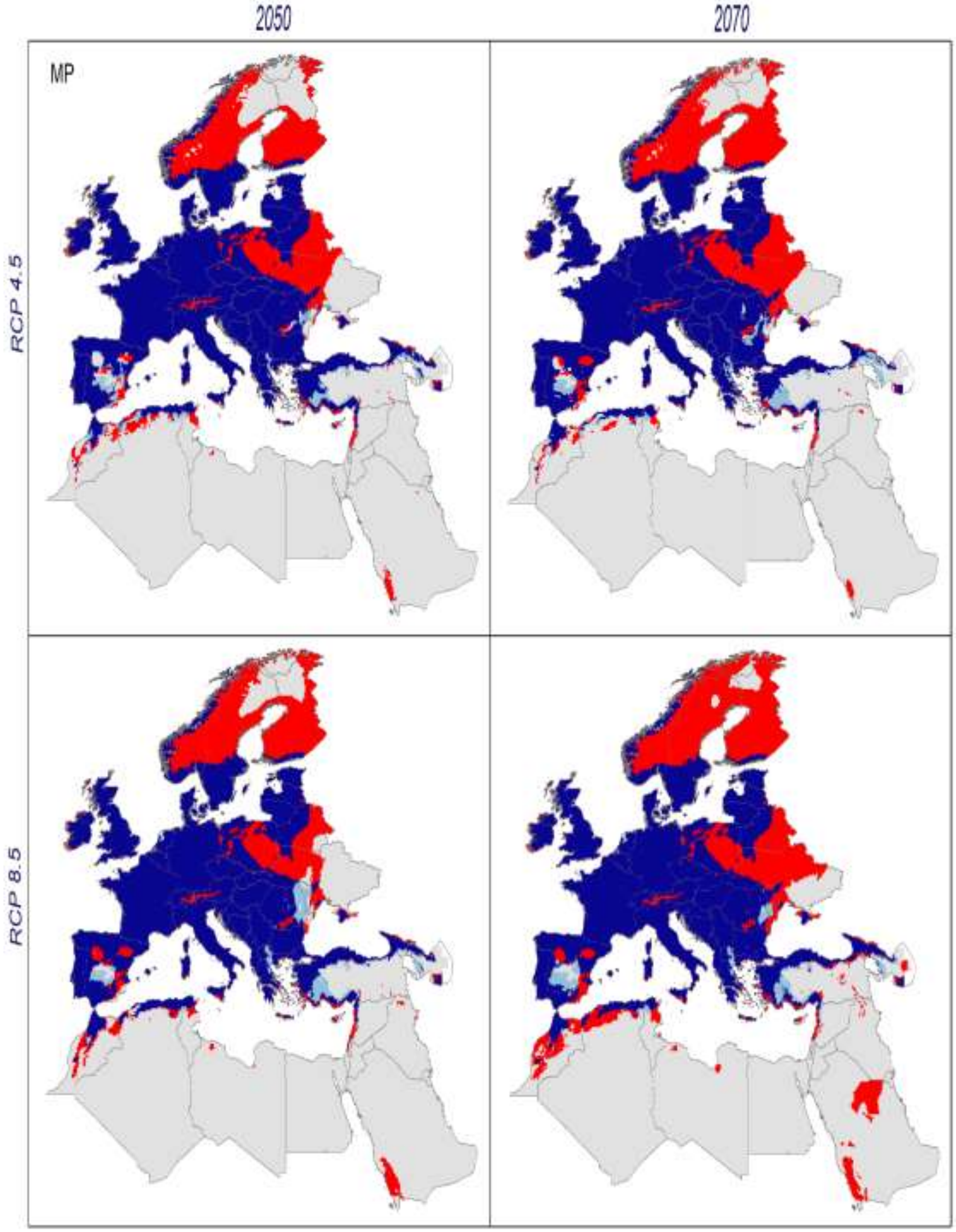

16. The predicted potential distribution based on the GCM of the Japan Agency for Marine-Earth Science and Technology, Atmosphere and Ocean Research Institute (The University of Tokyo), and National Institute for Environmental Studies (MIROC – ESM).

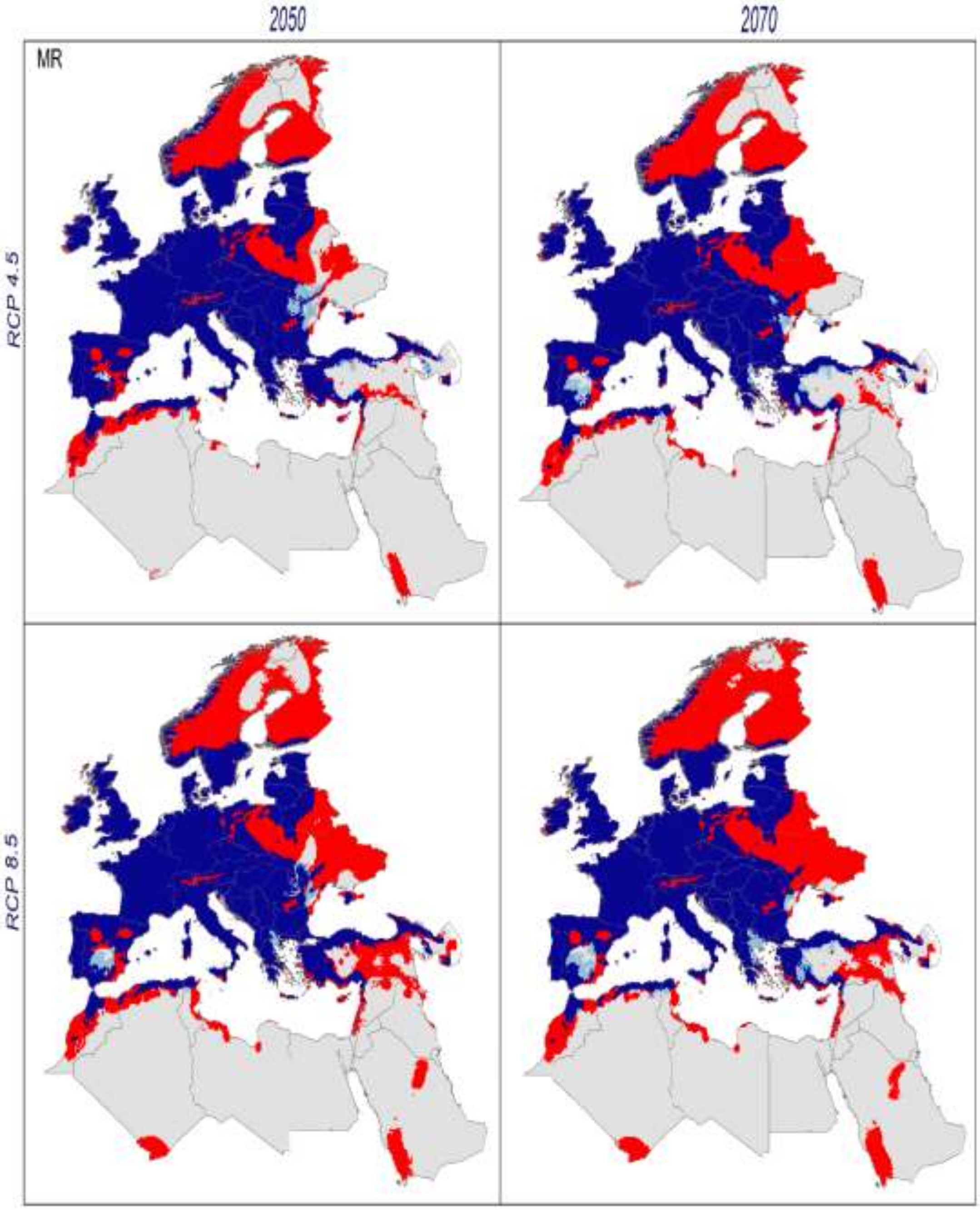

17. The predicted potential distribution based on the GCM of the Norwegian Climate Centre, Norway (NorESM 1 – M).

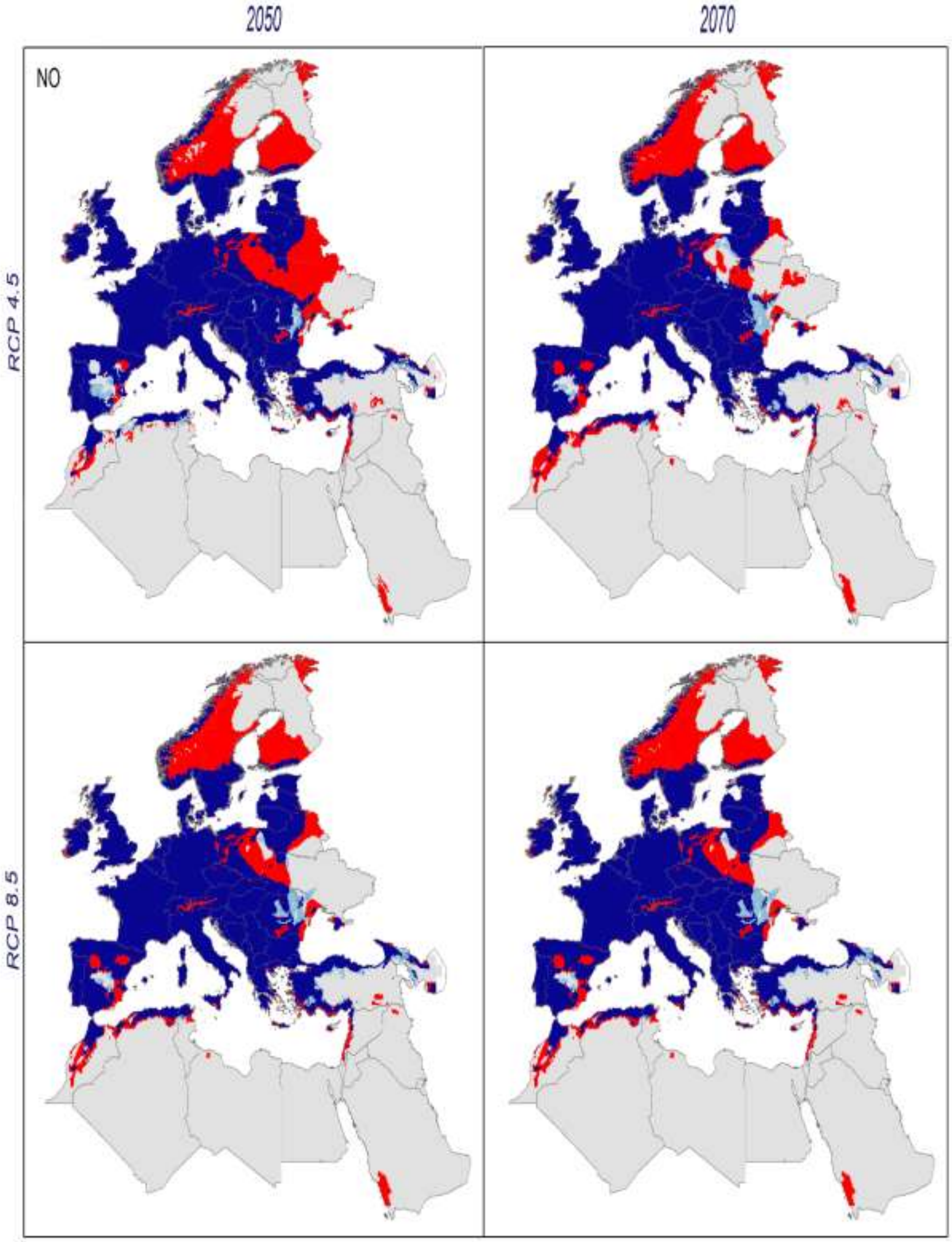

Supplement: S6 File — (PDF) [file pone.0189092.s006.pdf]
